# The contribution of environmental and dispersal filters on phylogenetic and taxonomic beta diversity patterns in Amazonian tree communities

**DOI:** 10.1007/s00442-021-04981-0

**Published:** 2021-07-29

**Authors:** Juan Ernesto Guevara Andino, Nigel C. A. Pitman, Hans ter Steege, Manuel Peralvo, Carlos Cerón, Paul V. A. Fine

**Affiliations:** 1 Grupo de Investigación en Biodiversidad, Medio Ambiente y Salud-BIOMAS-Universidad de las Américas, Campus Queri, Quito, Ecuador; 2 Keller Science Action Center, The Field Museum, 1400 South Lake Shore Dr., Chicago, IL 60605-2496 USA; 3 Naturalis Biodiversity Center, Vondellaan 55, Postbus 9517, 2300 RA Leiden, The Netherlands; 4 Consortium for the Sustainable Development of the Andean Ecoregion (CONDESAN), Andean Forest Program, German Aleman E12-123 and Carlos Arroyo del Río, Quito, 170504 Ecuador; 5 Escuela de Biología Herbario Alfredo Paredes, Universidad Central, Quito, Ecuador; 6 Department of Integrative Biology, University of California, Berkeley, CA 94720-3140 USA

**Keywords:** Lineages, Beta diversity, Environmental filters, Amazon, Biogeography

## Abstract

Environmental and dispersal filters are key determinants of species distributions of Amazonian tree communities. However, a comprehensive analysis of the role of environmental and dispersal filters is needed to understand the ecological and evolutionary processes that drive phylogenetic and taxonomic turnover of Amazonian tree communities. We compare measures of taxonomic and phylogenetic beta diversity in 41 one-hectare plots to test the relative importance of climate, soils, geology, geomorphology, pure spatial variables and the spatial variation of environmental drivers of phylogenetic and taxonomic turnover in Ecuadorian Amazon tree communities. We found low phylogenetic and high taxonomic turnover with respect to environmental and dispersal filters. In addition, our results suggest that climate is a significantly better predictor of phylogenetic turnover and taxonomic turnover than geomorphology and soils at all spatial scales. The influence of climate as a predictor of phylogenetic turnover was stronger at broader spatial scales (50 km^2^) whereas geomorphology and soils appear to be better predictors of taxonomic turnover at mid (5 km^2^) and fine spatial scales (0.5 km^2^) but a weak predictor of phylogenetic turnover at broad spatial scales. We also found that the combined effect of geomorphology and soils was significantly higher for taxonomic turnover at all spatial scales but not for phylogenetic turnover at large spatial scales. Geographic distances as proxy of dispersal limitation was a better predictor of phylogenetic turnover at distances of 50 < 500 km. Our findings suggest that climatic variation at regional scales can better predict phylogenetic and taxonomic turnover than geomorphology and soils.

**Supplementary Information:**

The online version contains supplementary material available at 10.1007/s00442-021-04981-0.

## Introduction

The longstanding debate concerning the role of ecological interactions and environmental filters vs. neutral processes as mechanisms explaining tree community assembly in Amazon forests is a prominent topic in ecological and evolutionary research (Tuomisto et al. 2002; ter Steege et al. 2006; Kraft et al. 2011; Swenson et al. 2013; Baker et al. 2014; Pos et al. 2019). Amazonian forests are among the most diverse in the world harboring around 16,000 tree species with some local communities containing over 300 tree species in a single hectare (ter Steege et al. 2013; Guevara et al. 2019; ter Steege et al. 2019; ter Steege et al. 2020). Thus, describing and analysing the patterns of species and lineage composition across spatial and environmental gradients is fundamental to understand the mechanistic causes that promote community assembly of this hyper-diverse ecosystem.

Both climate and soils are major environmental filters for Amazonian plant communities and are likely drivers of taxonomic turnover and phylogenetic beta diversity patterns among tree communities across the region (Antonelli et al. 2009; Fine and Kembel 2011; Honorio Coronado et al. 2015; Baldeck et al. 2016; ter Steege et al. 2006). It has been suggested that the heterogeneity of Amazonian soil types as a result of their complex geological history should be the main predictor of plant taxonomic turnover at local, landscape and regional scales in Amazonia (Terborgh and Andressen 1998; Tuomisto et al. 2002; Phillips et al. 2003; Higgins et al. 2011; Tuomisto et al. 2016). This pattern is attributed to differences in geology among regions (ter Steege et al. 2006; Pitman et al. 2008; Tuomisto et al. 2016). However, few studies have evaluated the combined effect of geology, soil nutrient availability, regional climatic variables and dispersal limitation on the patterns of phylogenetic beta diversity of Amazon tree communities. Such a comprehensive approach would allow us to investigate the historical and evolutionary processes that underlie the patterns of phylogenetic composition among tree communities at regional scales (Fine and Kembel 2011).

The role of geomorphology has been addressed in previous studies suggesting that Amazonian forests are partitioned into large floristic units associated with different geomorphological properties (Higgins et al. 2011; Tuomisto et al. 2016). Therefore, broad scale differences in soil composition should lead to significant and abrupt changes in plant species composition across landscapes (Mallet 2008; Fine et al. 2013; Tuomisto et al. 2016). Specifically, contemporary correlations between plant composition and geological and edaphic patterns in western and central Amazonia are thought to be driven by transitions from nutrient-rich Miocene–Pleistocene sediments in areas close to Andean foothills to the nutrient-poor Pliocene–Pleistocene sediments that lie to the east of the basin (Rossetti et al. 2005; Higgins et al. 2011).

The role of climate in tree species distribution has been recently addressed by research showing that precipitation gradients are associated with changes in species in tropical tree communities, including Amazonian forests (Hardy et al. 2012; Esquivel-Muelbert et al. 2016). For example, Esquivel-Muelbert et al. (2016) proposed that a large proportion of tree species in the Western Neotropics are affiliated with ever-wet conditions and, therefore, have ranges restricted to the wet extreme of the climate gradient, whereas other species of trees appear to be restricted to dry environments. Furthermore, a gradient in dry season length from western to southeastern Amazonia was found to be correlated with geographic variation in tree species composition across this longitudinal gradient (ter Steege et al. 2006). These results corroborate the idea that climate might act as the main environmental filter determining the regional species pool (Engelbrecht et al. 2007; Pennington et al. 2009; Lessard et al. 2011, 2012).

Two alternative hypotheses about the drivers of tree community assembly related to past climates have been recently put forward. The first proposes that Andean orogeny may promote lineage divergence via allopatric speciation and niche conservatism in areas close to the Andes (e.g. Western Amazonia) that have experienced potential climatic stability (limited beta niche evolution) since the Miocene until the Quaternary (Ackerly 2006; van der Hammen and Hooghiemstra 2000; Antonelli et al. 2009; Hoorn et al. 2010). The second argues about a fundamental role of recent Quaternary climatic changes promoting species diversification either via allopatric or parapatric speciation (Carnaval et al. 2009; Sandel et al. 2011). Thus speciation by local adaptation related to beta niche evolution might be prevalent across spatio-environmental gradients (Ackerly 2006; Graham et al. 2009; Eiserhardt et al. 2013).

Finally, evidence suggests that dispersal limitation is a fundamental driver of taxonomic turnover across spatial gradients (Condit et al. 2002; Pennington and Lavin 2016). The idea of dispersal-limited communities in Hubbell’s Neutral Theory of Biodiversity and Biogeography (NTBB), implies that all individuals of all species are ecologically equivalent and share the same probability of occupying a local assemblage. Ecological equivalence is the cornerstone of Hubbell’s Neutral Theory assuming that species members of a local community have identical average fitness and stabilizing mechanisms are completely absent (Hubbell 2001; Adler et al. 2007). Under this scenario, tree community composition may be the result of tree species’ dispersal abilities coupled with demographic stochasticity (Hubbell 2001). Thus, by increasing dispersal limitation among local communities that are part of a larger metacommunity, greater isolation of the local communities is expected. Isolation not only increases extinctions through demographic stochasticity but also differences in species composition among communities (beta diversity).

One of the main constraints in investigating the relative importance of geomorphology, soils, climate and dispersal limitation on beta diversity of Amazon tree communities is the lack of systematic sampling of tree communities along geomorphological units, and soil and climate gradients. Furthermore, few studies have included phylogenies at the community level to understand the historical and evolutionary processes that underlay the patterns of phylogenetic beta diversity among Amazon tree communities at regional scales (but see Fine and Kembel 2011; Honorio Coronado et al. 2015; Dexter et al. 2017). However, we can gain insights into the different biogeographical histories of regional species pools by investigating how phylogenetic relatedness among tree communities changes across environmental and spatial gradients (Graham and Fine 2008). Phylogenetic beta diversity (PBD) and taxonomic beta diversity patterns (TBD) can be evaluated on the basis of its turnover and nestedness components (Baselga 2010; Leprieur et al. 2011). Whereas the nestedness component of beta diversity is associated with gain or losses in species and lineages produced by density dependent factors or limited niche evolution (Baselga et al. 2007; Leprieur et al. 2011), the turnover component is related to the spatial replacement of some species and lineages by others caused by environmental filtering and dispersal limitation. In this paper we expand previous tests of the role of geology, geomorphology, soils, climate and dispersal limitation as drivers of taxonomic and phylogenetic turnover in Amazonian tree communities (Fine and Kembel 2011; Higgins et al. 2011; Honorio Coronado et al. 2015; Tuomisto et al. 2016; Cardenas et al. 2017). We address two main questions:

1. To what extent does spatial variation in climate, soils, geology and geomorphology drive patterns of phylogenetic and taxonomic turnover?
2. At what spatial scale is the role of climate, geomorphology and soils most important as an environmental filter for tree community composition?

To answer these questions, we posited the following hypotheses about the role of the aforementioned environmental filters and dispersal limitation as drivers of phylogenetic and taxonomic turnover. If climate is the main driver of community assembly of Amazon tree communities at biogeographic scales (H1) we predict climatic differences to be significantly associated with phylogenetic and taxonomic turnover at broad spatial scales. In addition, patterns of high taxonomic and high phylogenetic turnover with respect to climatic distances may be indicative of longstanding and disparate evolutionary histories among communities (limited beta niche evolution). Limited climatic niche evolution may result in patterns of increasing phylogenetic clustering as climatic distance increases.

If soils and geomorphology play a fundamental role in taxonomic and phylogenetic composition at biogeographic scales as previous studies have reported; (H2) we should expect a strong and significant association of taxonomic and phylogenetic turnover with geomorphological and soil variables operating at broad spatial scales. High taxonomic and high phylogenetic turnover mediated by incremental edaphic differences may be the result of pervasive habitat specialization of closely related species to broad spatial scale geomorphological or soil variables (phylogenetic clustering) (H3). We should expect high taxonomic turnover and low phylogenetic turnover if strong differences in soil composition at fine spatial scales causes closely related species to occur in contrasting edaphic habitats. Finally, if dispersal limitation is pervasive on taxonomic and phylogenetic turnover patterns (H4) we should expect a strong association of both phylogenetic and taxonomic turnover as geographic distance increases. Thus, high taxonomic and high phylogenetic turnover at large spatial scales may be the result of strong dispersal limitation determined by similar dispersal capabilities shared by close relatives. This effect should be stronger than the environmental filters (i.e. climatic, geomorphological and edaphic gradients) causing an increase in phylogenetic clustering as geographic distance increases.

## Materials and methods

### Study site

To study the relationships of geomorphology, soils, climate and geographic distances with patterns of phylogenetic and taxonomic turnover we studied tree communities in the Ecuadorian Amazon located on a gradient of climatic, edaphic and geomorphological conditions. The landscape is mostly dominated by rolling hills interrupted by terrain depressions or *baixios* that vary in extent and levels of drainage (Pitman 2001). Terraces of Pleistocene origin dominate the northern and southern banks of the Aguarico River, whereas the northern bank of the Napo River is mainly covered by palm-dominated swamps (Ministerio de Ambiente del Ecuador 2013). The Pastaza River represents a significant geomorphological break in the landscape of the Ecuadorian Amazon. South of this river the landscape is characterized by extensive plains of *terra firme* forests interspersed by swamps that are often dominated by palms. This area is known as the Pastaza Fan, a massive volcanoclastic alluvial fan deposited during the Holocene (Rasanen et al. 1987; Bernal et al. 2011). Finally, we sampled the lowland forests adjacent to the Cordillera del Cóndor, which is one of the areas of Ecuadorian Amazon that remains poorly explored in terms of floristic inventories. We sampled one plateau on quarzitic sandstones that represents the lowest elevations of Cordillera del Cóndor. The main geological unit of this area is the Tena Formation, which has been dated to the Cretaceous (Lee et al. 2004).

### Tree community sampling

**Fig. 1:**
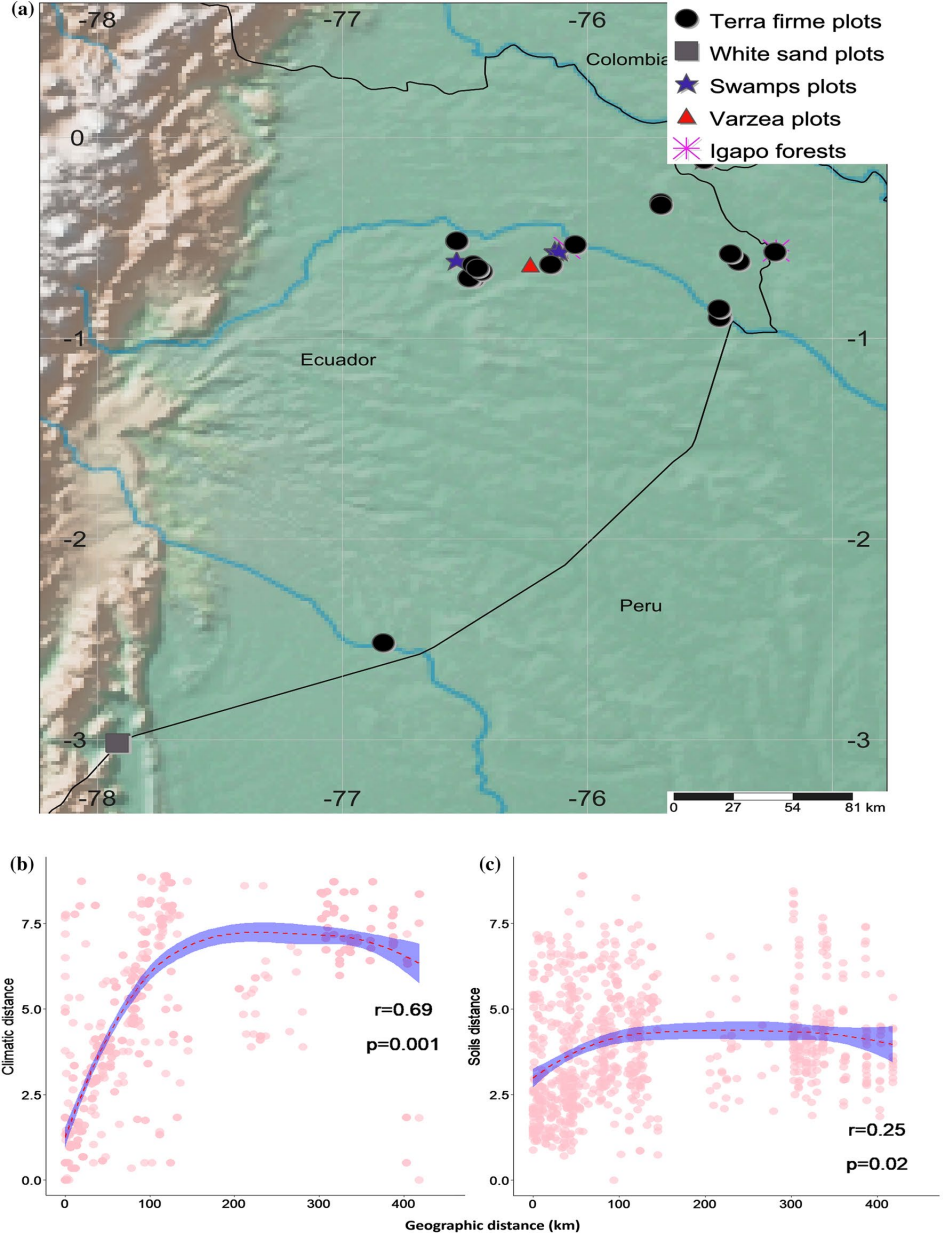
**a** Map of the study site, showing the geological map of the Ecuadorian Amazon overlain on a digital elevation model (STRM) of the region. Symbols and colors represent the plot locations and the results of non-metric multidimensional analysis based on a phylogenetic dissimilarity matrix (from Guevara et al. 2017). Correlations between **b** climate and **c** soils with respect to geographic distance as an indicative of the environmental and spatial gradients of the study area

We established a network of 41 one-hectare plots distributed across the Ecuadorian Amazon in native undisturbed forests. This network includes 26 plots in *terra firme* forests, 5 plots in white sand forests, 5 in swamps, 4 in varzea forests and 1 in *igapó* forests (Pitman et al. 2001; Guevara et al. 2017; ter Steege et al. 2013). (Fig. 1). Our plot network spans 2 degrees in longitude and 1.5 degrees in latitude, an appropriate geographic scale to detect the effect of environment and geographic distance on the phylogenetic and taxonomic turnover patterns of Amazon tree communities.

In each one-hectare plot we recorded, tagged and identified all trees with diameter at breast height (dbh) ≥ 10 cm. This dataset includes 34,874 individual trees. Herbarium specimens for every tree species were collected and duplicates deposited and compared with botanical specimens from four herbaria (QCNE, QCA, QAP, F). We standardized the taxonomy of the vouchers collected in this study. When possible, we confirmed the identification of species collected in the field with taxonomic specialists in each group. Most of the specimens we collected were sterile (80%). We are aware this might represent a limitation because taxonomic specialists not usually consider this material for species names confirmation. However, in the past 5–8 years the number of fertile material for many of our vouchers have been collected by the authors or other researchers and the taxonomy of the plot network verified (Guevara et al. 2019). In many other cases our extensive experience in Amazonian tree species identification gives us confidence in the accuracy of the taxonomy in our plot network.

Finally, we excluded morphospecies that were not possible to be classified at genus level from the phylogenetic and statistical analyses. We excluded 98 morphospecies that were not classified at genus level and from this number 20 morphospecies were unable to be assigned to any taxonomic family. This number represents approximately 5% of the total number of species recorded in our data set and, therefore, unimportant to affect the results of the ecological patterns we investigated.

### Phylogenetic tree

We created a phylogeny for 1,687 operational taxonomic units (OTUs) (Figure S4, Appendix S1) using as a backbone tree a consensus maximum likelihood molecular phylogeny for 852 Amazon tree genera (Neves et al. 2020). This molecular phylogeny represents the most updated phylogenetic reconstruction of Amazon tree lineages based on rbcl and matK genetic markers (but see Dexter and Chave 2016). Tree topology and divergence times of taxa were estimated using a Bayesian Markov Chain Monte Carlo model, branch lengths were time-scaled using a relaxed molecular clock with fossil-based age constraints implemented on 86 nodes (Magallón et al. 2015). Then we used the web version of Phylomatic (Webb and Donoghue 2005) to graft the 1687 tree species using as input the list of tree species obtained from our floristic inventory. From this list we omitted unnamed species to diminish the effect of taxonomic uncertainty in the number of tips that belong to a particular lineage. Because an unnamed species may represent a morphological variant of a species but not a completely different lineage, artificial imbalance in branching patterns can be introduced due to poor taxonomy of a particular group or clade. Thus, a regional phylogeny of 1687 terminal nodes (species) and 561 internal nodes (genus) was used in the subsequent analysis.

While we are aware that our phylogenetic tree is not fully resolved at terminal nodes and that few new regional phylogenies at species level for the neotropical tree flora have been published recently (Coelho et al. 2019; Neves et al. 2020), we argue that the resolution of our phylogeny is sufficient to test the hypotheses we propose in this study. Mounting evidence suggests that unresolved terminal nodes (species) in a phylogenetic tree may have minor effects on detecting macro-ecological patterns and phylogenetic composition differences at deeper nodes (Swenson 2009). In addition, Swenson (2009) demonstrated that unresolving most terminal nodes in the phylogeny have much less influence on the power to predict NRI and NTI values. This is particularly important when detecting non-random patterns of phylogenetic dispersion among pairs of communities. For instance, large phylogenies with many unresolved terminal nodes but with most of the basal nodes fully resolved are less prone to be biased towards reduced statistical power to detect non-random phylogenetic community structure (in the case of our study beta NRI and beta NTI). While this loss of power needs to be considered when interpreting results, the evidence from previous works also suggests that this effect is minimized using supertrees with fully bifurcating basal nodes (Swenson 2009).

However, for comparison purposes we generated a fully resolved phylogeny of 931 species and compared the observed values of phylogenetic turnover derived from this tree with observed values derived from the genus level phylogeny (Figure S1). We also estimate the relationship between climatic and soils distance with respect overall phylogenetic beta diversity using the 931 species phylogeny generated from the phylogenetic tree published by Neves et al. 2020 (Figure S1).

### Phylogenetic and taxonomic turnover

The change in phylogenetic and species composition among local communities was measured calculating species and phylogenetic turnover (turnover component), and overall change in both lineages and species composition (overall beta diversity) (Baselga 2012; Leprieur et al. 2012). We calculate the turnover component of beta diversity as follows:

$${\text{Sorenson}}\left( {{\text{tur}}} \right) = \frac{{[\Sigma {\text{ min}}(bij,\;bji)]\; + \;\Sigma {\text{max}}(bij,\;bji)]}}{{2\left[ {\Sigma\; Si - ST} \right] + [\Sigma {\text{min}}(bij,\;bji)]\; + \;\Sigma {\text{max}}(bij,\;bji)]}},$$

where *S*_*i*_ is the total number of species in community *i* and S_T_ is the total number of species considering all communities together and *b*_*ij*_ and *b*_*ji*_ the number of species present only in sites *i* and *j*, respectively, when these sites are compared by pairs.

The phylogenetic version of the turnover component of Sorenson index was calculated as follows:

$${\text{Phylosorenson}}({\text{tur}}) = \frac{{({\text{PD}}_{{{\text{Tot}}}} - {\text{PD}}_{i} ,{\text{PD}}_{{Tot}} - {\text{PD}}_{j} )}}{{{\text{PD}}_{i} + {\text{PD}}_{j} - {\text{PD}}_{{{\text{Tot}}}} + 2{\text{min}}({\text{PD}}_{{{\text{Tot}}}} - {\text{PD}}_{i} ,{\text{PD}}_{{{\text{Tot}}}} - {\text{PD}}_{j} )}},$$

where, PD_tot_ is the sum of branch length common to both communities *j* and *k*, and PD_*j*_ and PD_*i*_ are the sum of branch lengths that are present in community *j* but not in community *i* and the sum of branch lengths present in the assemblage *i* but not found in community *j*, respectively.

Finally, overall phylogenetic beta diversity (PBD) was calculated as follows:

$${\text{Phylosorenson}}({\text{PBD}}) = \frac{{2{\text{PD}}_{{{\text{Tot}}}} - {\text{PD}}_{i} - {\text{PD}}_{j} }}{{{\text{PD}}_{i} + {\text{PD}}_{j} }},$$

where, PD_Tot_ is total sum of branch length for both communities *j* and *i*, and PD_*i*_ and PD_*j*_ are the sum of branch lengths of community *i* and *j*, respectively.

Overall taxonomic beta diversity (TBD) was calculated as follows:

$${\text{Sorenson}}\left( {{\text{TBD}}} \right) = \frac{{{\text{b}}_{i} + {\text{c}}_{j} }}{{\left( {2a_{{ji}} + b_{i} + c_{j} } \right)}},$$

where *a*_*ji*_ is the fraction of species shared in communities *i* and *j* and *b*_*i*_ and *c*_*j*_ are the proportion of species just present in communities *i* and *j,* respectively.

We compared the observed values against the expected values of taxonomic turnover using a null model that produces random draws from the regional pool (here defined as the total number of species in our plot network). This model maintains species richness for each local community and the number of species shared between communities with equal probability to colonize them. To compare the observed values against the expected values of phylogenetic turnover we used a null model that assumes all species in the regional phylogeny have equal probability of colonizing a local community in such a way that dispersal limitation or long-distance dispersal has only minor effects on the assembly of communities. Thus, when interpreting the results, we infer that pairs of compared communities are composed of lineages that are closely related if the observed values of phylogenetic turnover (PT) are less than the expected values based on the null model. To evaluate whether patterns of high phylogenetic turnover are consistent with an increase in phylogenetic clustering as differences in environment and space increases, we performed an additional analysis to evaluate terminal and basal phylogenetic beta diversity (Swenson 2011). We used the standardized effect size for the mean nearest taxon distance among taxa in different communities (beta NTI) to assess phylogenetic clustering at the tips of the regional phylogenetic tree (terminal phylogenetic beta diversity) (Webb 2000; Fine and Kembel 2011). To evaluate phylogenetic clustering at deeper nodes of the regional phylogeny (basal phylogenetic beta diversity) we used the standardized effect size of the mean pairwise phylogenetic distances among taxa in different communities (beta NRI) (Webb 2000). To test whether the patterns deviate from the null expectation of phylogenetic beta diversity patterns we performed a null model that makes random draws without replacement from the full list of species present in the phylogeny pool. In this model all the species in the phylogeny have equal probability of being included in the null communities. We used the inverse of these metrics (see Dexter et al. 2017), thus positive values of beta NRI and beta NTI are interpreted as evidence for phylogenetic evenness among different communities towards the tips and deeper nodes of the phylogenetic tree. Negative values of beta NRI and beta NTI are interpreted as evidence of phylogenetic clustering among different communities.

### Climatic variables

To assess the role of climatic variables in the patterns of taxonomic and phylogenetic turnover we used 19 climatic variables from Worldclim at 30 s of resolution as an initial set of variables (Table S1.1). We then performed a forward selection procedure to select significant variables that were used in further analysis (see details in Sect. “Statistical analysis”).

### Geomorphological variables

To evaluate the role of geomorphology as predictor of phylogenetic and taxonomic turnover, we used a digital elevation model (DEM) for the Ecuadorian Amazon obtained from the Shuttle Radar Topography Mission (SRTM) and distributed by the USGS through the Earth Explorer platform (https://earthexplorer.usgs.gov/). We created a consensus map by overlaying the rasterized geological map with the DEM using the sum option in the ArcGIS 10.3 software. We subsequently generated geomorphological indices to use in subsequent analysis. Geomorphological variables provide us general information about the origin and timing of parent material formation and topography and, therefore, we use it as surrogate for historical events. Four variables (hierarchical slope position, slope, dem and landsat) were used in the analysis describing the geomorphology and land cover features in the vicinity of the forest plots Hierarchical Slope Position identifies topographic exposure (ridge, slope, valley bottom, etc.), while slope position indicates that the central point is located higher or lower than its average surroundings. Digital elevation models (DEM) measure the bare-earth surface based on raster grids of the elevation between two or more points. Land cover information was obtained from a mosaic of Landsat images for the period 2010–2014 that had been created for the Ministry of the Environment of Ecuador (see details for calculations in Appendix S1). All geomorphological variables were extracted from the elevation data using the Spatial Analyst extension in ArcGIS 10.3 software from ESRI (Environmental Systems Resource Institute).

### Soil variables

Soil samples from the 41 one-hectare plots were taken from the four corners and the centre of each plot, then dried separately and subsequently mixed to obtain a single sample per plot. We measured nine edaphic variables: pH, organic matter (%), sand (%) silt (%) and clay (%), P, Ca, Mg and K all measured as part per million (ppm). Non-nitrogen elements were extracted with Mehlich-III solution and analysed on an atomic emission-inductively coupled plasma (AE-ICP, Perkin Elmer Inc., Massachusetts, USA). Nitrogen was extracted following the methods in Baldeck et al. (2016). Soil texture was measured in percentage sand, clay and silt and cation content was measured in parts per million (ppm) (Table S.1). All soil analyses were done in the laboratory of the Facultad de Geologia, Minas y Petroleo (Labgeimpa) of the Universidad Central del Ecuador.

### Statistical analysis

Prior to analysis, soils and climatic variables were square root transformed and geomorphological variables were standardized adding the cubed values of each variable according to the methods of Legendre et al. (2015). We performed this standardization previous subsequent analysis because the scaling we used to measure each environmental variable might not be the most relevant scaling to understand how these variables determine species composition and distribution. Therefore, we do not know how species responds to environmental variation and thus we need to reduce the bias introduced by the different scale units we used when measuring soils and climate variables.

### To what extent does spatial variation in climate, soils, geology and geomorphology drive patterns of phylogenetic and taxonomic turnover?

To evaluate the role of environmental and dispersal filters as determinants of taxonomic and phylogenetic turnover we followed a two-step process. First, we described patterns of variation in both taxonomic and phylogenetic composition across geomorphology, soils, climate and spatial distances. For this purpose, we performed a Non-Metric Multidimensional Scaling analysis (NMDS), using taxonomic and phylogenetic turnover matrices as input. We used the first two dimensions in the ordination and 1000 random starting iterations to obtain the lowest stress value that determines the best solution for that ordination, we carried out this step using the function metaMDS and the argument try = 1000 with the package “vegan” in the statistical programme R (R Development Core Team 2011). Then we tested the explanatory power of environmental and spatial variables using a distance-based redundancy analysis (db-RDA) (Legendre and Anderson 1999). We performed forward selection procedure for the full set of environmental variables using 9999 permutations and an alpha value of 0.05 to set the statistical significance of the selected variables. In this analysis, each new variable added to the model had to achieve an α of 0.05 and the cumulative adjusted *R*^2^ of the model could not exceed the adjusted *R*^2^ of the model created from all variables (Baldeck et al. 2016). From the initial set of 32 environmental variables, we obtain 18 environmental variables that we used in the subsequent analyses. We performed a forward selection procedure of these 19 climatic variables, selecting 13 final variables that were used to create climatic dissimilarity matrices. To incorporate the full set of climatic variables we performed a Principal Component Analysis using a correlation matrix to avoid collinearity among the variables. We decided to use this approach instead of selecting only those climatic variables exhibiting high correlations to avoid missing valuable information. Finally we selected the two first axes of the PCA that explained most of the variation, 65.29% and 20.56%, respectively.

This technique works on the basis of traditional RDA but allows the incorporation of any non-Euclidean distance measurement. In our db-RDA, dissimilarities matrices of taxonomic and phylogenetic composition, based on Sorensen and Phylosorensen indexes, were used to perform a Principal Coordinates Analysis, and all the positive eigenvalues were retained for further analysis. The eigenvalues were used as the response matrix in the traditional RDA. In comparison with the widely used Mantel test on distance matrices db-RDA has some advantages; (1) it allows us to incorporate any non-Euclidean distance measure, (2) it uses non parametric permutation methods that do not assume multivariate normality and (3) contrary to a Mantel test, canonical redundancy analysis correctly estimates the proportion of the original data variation explained by spatial structures. Finally, we compared environmental and geographic distances with phylogenetic dissimilarity matrices to assess the role of environmental and dispersal filters as drivers of phylogenetic beta diversity patterns. In this step, we used as input for phylogenetic dissimilarity the turnover component of Sorensen index and beta NRI and beta NTI.

### At what spatial scale is the role of climate, geomorphology and soils most important as an environmental filter for tree community composition?

Second, to analyse the spatial scale at which each environmental and spatial predictor is most important as a driver of phylogenetic and taxonomic turnover we used principal coordinate neighbour matrices (PCNM) to decompose the pure spatial relationships between plots, the spatial variation in environmental variables and the unique contribution of environment (Borcard et al. 2004; Legendre et al. 2009; Peres-Neto and Legendre 2010). We decided to use Principal Coordinates of Neighbourhood Matrices analysis due to the inherent spatially autocorrelated structure of geomorphological, climatic and soil variables. PCNM eigenfunctions represent the spectral decomposition of the spatial relationships between plots, therefore, describing all possible spatial scales that can be defined on the basis of geographical distances between plots (Legendre et al. 2009). In addition, this analysis allowed us to determine the spatial scale at which the response data (e.g. environmental variables) were spatially structured and, therefore, identify the relationships species-environment at these relevant scales (Borcard et al. 2004). Then, we performed a forward selection procedure on the PCNM table to determine if the spatial structure was mostly broad-, mid- or fine-scaled, (50, 5, or 0.5 km^2)^ following Legendre et al. (2009) and Peres-Neto and Legendre (2010). After this procedure, we selected PCNMs 1, 2, 10, 11, 24 and 28 that were found to determine broad, mid and fine spatial scales, respectively, at the 5% significance level. The rest of PCNMs were not considered in the analysis due to their lack of statistical significance (Table S3).

The variation in both phylogenetic and taxonomic composition of the tree communities was partitioned with respect to climate, geographic distance, geomorphology and soils. To perform variation partitioning analysis, we used Canonical Redundancy Analysis (RDA) using as input PCA axis 1 for each environmental variable and derived from the full set of climate, soils and geomorphological variables. As a measure of phylogenetic and taxonomic turnover we used axis 1 and 2 of a NMDS analysis based on taxonomic and phylogenetic dissimilarity matrices. We decided to use the full set of environmental variables instead of omitting those variables exhibiting high correlations to avoid missing valuable information.

First, we evaluated the relative contribution of each environmental variable (soils, geomorphology and climate) by partitioning the total phylogenetic and compositional variation explained by each environmental variable and the full environmental data set. Second, we assessed variation by partitioning the total phylogenetic and compositional variation explained by the interaction of geomorphology-soils, climate-spatial distance, soils-climatic distance, geomorphology-spatial distance and spatial variables using PCNMs 1 and 2 (separately and in combination) defining broad spatial scales (50 km^2^), 10 and 11 defining mid spatial scales (5 km^2^) and PCNMs 24 and 28 defining fine spatial scales (0.5 km^2^) were used in each model accounting for each environmental factor. We decided to use this approach because PCNM eigenvectors allow us to assess the patterns of taxonomic and phylogenetic turnover at multiple spatial scales, while RDA analysis allow us to account for the unique contribution of climate, geomorphology, soils, spatial distances.

Principal coordinate neighbour matrices analysis was computed with the “spacemakeR” and the PCNM libraries in the R statistical language (Dray et al. 2013). Forward selection of PCNM eigenfunctions, geomorphological, edaphic and climatic variables was performed with the “packfor” library (Lichstein 2007). To create the regional phylogeny, we used the “picante” package in R (Kembel et al. 2010; Webb et al. 2008). Phylogenetic and Taxonomic turnover analyses were carried out with the “betapart” (Baselga 2012) and “vegan” (Oksanen et al. 2015) packages (R Development Core Team 2007). Redundancy analyses were carried out with the “vegan” (Oksanen et al. 2015) package.

## Results

### The influence of climate, soils-geomorphology and dispersal filters on phylogenetic turnover

The results of the db-RDA showed that 36% of variation in phylogenetic turnover was explained by the constrained axis 1 of the ordination, the second constrained axis explained 18% of the total variation in phylogenetic composition. The cumulative proportion of variance explained by the two first constrained axes was 52% when phylogenetic turnover was taking into account. The most important variables in the model for phylogenetic turnover were annual precipitation, temperature seasonality, DEM 0.5 km and P (phosphorous) which were highly correlated with the first and second axes (Table 1 and Figurer 3A). The first axis of described a gradient in soils fertility specially for Mg, K, silt and sand whereas the second axis was strongly influenced by a gradient in climate and geomorphology especially for temperature and precipitation. The forward selection procedure demonstrated which individual variables from the full set of environmental variables contributed significantly to explain phylogenetic turnover among plots (Table 2). There was a highly significant association between climate and phylogenetic turnover among tree communities in the Ecuadorian Amazon and this association was highly significant at all spatial scales (Table 2).

**Table 1:** Importance and significance of the constrained axes with positive eigenvalues in a distance-based redundancy analysis (db-RDA) model for phylogenetic and taxonomic turnover in Amazonian tree communities

Phylogenetic turnover					Taxonomic turnover			
	*df*	Sum of Squares	*F*	*Pr(*> *F)*	*df*	Sum of Squares	*F*	*Pr(*> *F)*
dbRDA1	1	1	138.231	0.001	1	23.068	99.442	0.001
dbRDA2	1	049057	61.653	0.016	1	10.649	45.905	0.025
dbRDA3	1	032987	41.457	0.018	1	10.079	43.449	0.001
dbRDA4	1	016162	20.311	0.975	1	0.4949	21.333	0.684

Only the four most important axes are shown, significance was assessed with a permutation test (1000 permutations) on the Pseudo-F

**Table 2:** Results of db-RDA analyses of phylogenetic and taxonomic turnover versus environmental variables among 41 one-hectare plots in Ecuadorian Amazon

Phylogenetic turnover					Taxonomic turnover				
Environmental variables	*df*	Sum of squares	*F*	*Pr(*> *F)*		*df*	Sum of squares	*F*	*Pr(*> *F)*
dem0.5 km	1	0.29186	36.680	0.001	dem0.5 km	1	0.8971	38.671	0.001
dem5km	1	0.08750	10.996	0.328	dem5km	1	0.2859	12.323	0.189
dem50km	1	0.10677	13.419	0.183	dem50km	1	0.2689	11.590	0.245
Orgmat	1	0.13033	16.379	0.080	Orgmat	1	0.3940	16.985	0.031
P	1	0.26299	33.052	0.001	P	1	0.5920	25.520	0.003
K	1	0.08305	10.437	0.348	K	1	0.3062	13.199	0.156
Ca	1	0.13054	16.406	0.073	Ca	1	0.3471	14.964	0.071
Mg	1	0.25207	31.679	0.004	Mg	1	0.6144	26.484	0.001
Sand	1	0.18182	22.851	0.012	Sand	1	0.4815	20.757	0.009
Silt	1	0.14657	18.421	0.055	Silt	1	0.3952	17.035	0.037
Clay	1	0.21721	27.298	0.009	Clay	1	0.4855	20.929	0.009
Temperature seasonality	1	0.41254	51.846	0.001	Temperature seasonality	1	0.9956	42.918	0.001
Annual precipitation	1	0.23001	28.906	0.006	Annual precipitation	1	0.5615	24.207	0.003
Precipitation driest month	1	0.12631	15.874	0.091	Precipitation driest month	1	0.3417	14.730	0.070
Precipitation coldest quarter	1	0.10231	12.858	0.184	Precipitation coldest quarter	1	0.2940	12.674	0.163
Precipitation seasonality	1	0.10714	13.465	0.160	Precipitation seasonality	1	0.3292	14.191	0.100
Precipitation wettest quarter	1	0.08640	10.858	0.314	Precipitation wettest quarter	1	0.2477	10.676	0.301
Precipitation warmest quarter	1	0.09565	12.021	0.255	Precipitation warmest quarter	1	0.2821	12.161	0.178

We found a weak but highly significant relationship between values of the nearest taxon index and environmental and dispersal filters, suggesting that the average pair of tree communities in the Ecuadorian Amazon were phylogenetically clustered towards the tips of the regional phylogeny (Fig. 4). Taxa in pairs of tree communities sharing similar climates were phylogenetically even towards the tips of the tree and taxa in pairs of tree communities that experienced different climates were phylogenetically clustered towards the tips of the regional phylogenetic tree (Fig. 4A). We found a non-significant correlation between net relatedness index and climatic distances and pairs of tree communities were randomly assembled with respect to climate. Soil distances among pairs of tree communities were significantly correlated with phylogenetic clustering towards the tips but not tree wide and this correlation was weaker than the effect of climatic distances on phylogenetic clustering towards the tips of the regional phylogenetic tree (Fig. 4). Taxa in pairs of tree communities that were close in space were phylogenetically even towards the tips of the tree and the effect of geographic distances determining phylogenetic clustering towards the tips of the regional phylogenetic tree was strongest at distances of 200–400 km (Fig. 4C). Furthermore, pairs of tree communities were also phylogenetically clustered tree wide at spatial distances of 200–400 km (Fig. 4F).

### Partitioning the influence of climate, soils-geomorphology and dispersal filters on phylogenetic turnover

We found that the effect of climate on phylogenetic turnover patterns was overwhelmingly more important at broad scales (41% of variation explained) than its effect at mid or fine spatial scales (Table 2). The fraction of total variation in phylogenetic composition explained by the combined effect of climate and spatial distances was significantly higher at broad spatial scales (19% of explained variation) compared to mid or fine spatial scales (Fig. 5b).

Geomorphology explained a large proportion of the variation in phylogenetic turnover at broad spatial scales but was a weak predictor at mid and fine scales; meanwhile soils explained a large proportion of phylogenetic turnover at all spatial scales (Table 2). There was a low and non-significant effect of the combined effect of soils and geomorphology on the explained variation of lineages composition at broad and mid spatial scales (3% and 6% of explained variation, respectively). The effect of this interaction explained 13% of the variation in phylogenetic turnover at fine spatial scales. We found a strong, significant effect of dispersal limitation on the variation in phylogenetic turnover when we analysed the fraction of variation in lineage turnover explained by spatial distances (Table 2).

### The influence of climate, soils-geomorphology and dispersal filters on taxonomic turnover

**Fig. 2:**
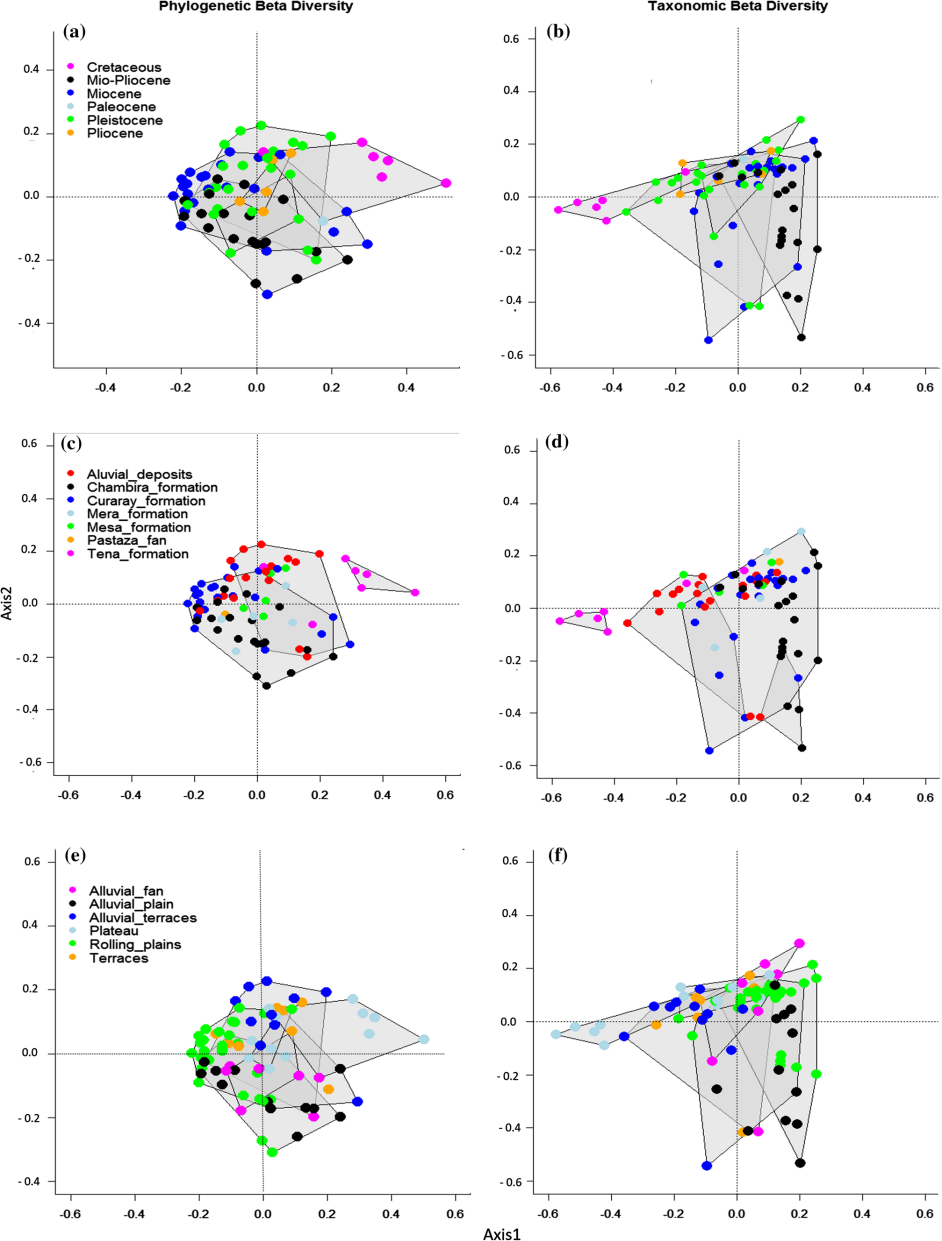
NMDS ordinations showing groups of phylogenetically and taxonomically similar plots based on the results of NMDS for **a**, **b** geological ages. **c**, **d** geological formations and **e**, **f** geomorphological features. Convex hulls represent 90% confidence intervals

**Fig. 3:**
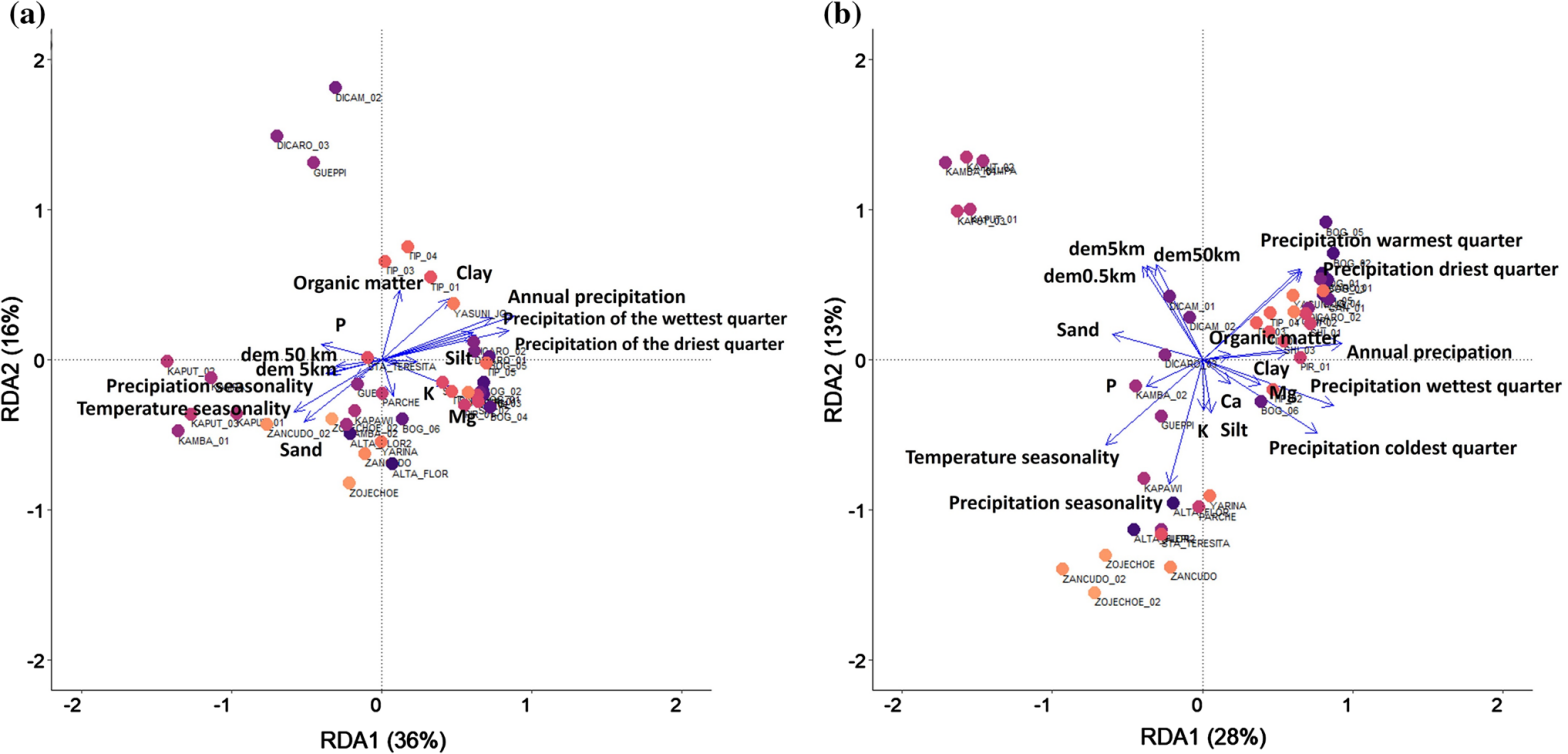
Results of a distance-based redundancy analysis (db-RDA) examining the relationship between phylogenetic and taxonomic turnover (represented using the turnover component of Phylosoreson and Sorenson indexes, respectively) and environmental variables selected by forward selection. **a** The first two axes of the db-RDA for phylogenetic turnover are shown and **b** the first and third axes of the db.RDA are shown for taxonomic turnover. The color of dots represents tree communities associated by phylogenetic similarity. Arrows show the magnitude and direction of environmental variables along the ordination axes

The results of the DEM-geology map and the NMDS ordinations revealed that, even though there is a correlation between geomorphology and turnover in taxonomic composition, similar phylogenetic composition can occur in different geomorphological units suggesting low phylogenetic turnover (Fig. 2). We found that tree communities located on the Cretaceous plateaus of the Cordillera del Cóndor represented tree communities that were phylogenetically and taxonomically strongly differentiated from the rest of those in the Ecuadorian Amazon (Fig. 2). Tree communities on alluvial terraces of the Aguarico and Napo rivers were also floristically distinct, in spite of significant overlap in composition and geology between these forests and those located on the southern bank of the Napo. These plots are located in areas that we identified as alluvial deposits from Quaternary origin. Plots located in areas such as Yasuní National Park, which corresponds to the Curaray Formation (Miocene origin), are both taxonomically and phylogenetically most similar to plots located in areas toward the south of Yasuní on Chambira or Mera formations, of Mio-Pliocene and Plio-Pleistocene origin, respectively. We also found low phylogenetic turnover for plots located in forests of the Pastaza megafan, characterized by rich soils derived from the Mera Formation (Pleistocene) when compared with tree communities located on the rolling plains and hilly areas of Yasuní (Miocene) (Fig. 2A, Table S4.1). A total of 28% and 13% of the total variation in taxonomic turnover was explained by axes 1 and 3 of the db-RDA, respectively. The cumulative proportion of variance in taxonomic turnover explained by the first and third constrained axes was 25%. The most important variables in the model for taxonomic turnover were temperature seasonality, DEM 0.5 km, Mg and sand, which were highly correlated with the first and third axes (Table 1 and Fig. 3B). The first axis described a gradient in climate and soils fertility specially for temperature seasonality, precipitation seasonality, P, K, Mg, silt and clay whereas the second axis was strongly influenced by a gradient in climate and geomorphology, especially for temperature and precipitation. The forward selection procedure from the full set of environmental variables showed that individual variables related to geomorphology and soils contributed significantly to explain taxonomic turnover among plots (Table 2).

### Partitioning the influence of climate, soils-geomorphology and dispersal filters on taxonomic turnover

The variation partitioning analysis via RDA determined that, when considered alone, climate explained the largest fraction of the variation in the patterns of taxonomic turnover (Table 3A). The effect of climate was stronger at mid and broad spatial scales than at small scales and when compared with the fraction of variation explained by geomorphology and soils, the effect of climate on patterns of taxonomic turnover was significantly more important at all spatial scales (Fig. 5a, Table 3A). The fraction of the total variation in tree species composition, explained by the climatic variation related to spatial distance, was significantly higher at fine spatial and mid spatial scales but very weak at broad scales (Table 3A). Geographic distance alone explained a large proportion of the variation in species composition as the results of the partition analysis demonstrated (Table 3). We also found that the effect of spatial distances explaining variation in species composition was significantly higher at broad scales compared with mid and fine spatial scales (Table 3A).

**Table 3 Table 3:** Variance partitioning results for phylogenetic and taxonomic turnover at three different spatial scales for different environmental variables as explained by positive eigenvectors selected through forward selection

	Space	Geomorphology	Climate	Soils	Soils and geomorphology	Climate and spatial distance	Soils and spatial distance	Geomorphology and spatial distance	Environment space
Phylogenetic turnover									
Fine scale	0.4	0.07	0.14	0.16	**0.13**	0.07	**0.27**	0.02	**0.35**
Mid scale	0.2	0.004	0.15	0.15	0.06	**0.17**	**0.22**	0.01	**0.37**
Broad scale	**0.33**	**0.34**	**0.41**	**0.32**	0.03	**0.19**	0.01	0.14	**0.51**
Taxonomic turnover									
Fine scale	0.03	0.06	**0.26**	**0.24**	**0.18**	**0.29**	**0.21**	0.11	**0.44**
Mid scale	0.01	0.03	**0.28**	**0.23**	**0.13**	**0.27**	**0.18**	0.04	**0.49**
Broad scale	**0.42**	**0.41**	**0.52**	**0.42**	**0.12**	**0.01**	0.01	0.01	**0.61**

Components are proportion of variance explained by each variable; Environment |space is the proportion of variance explained by the full environmental variables set after accounting spatial distances (see text for details). Only significant (*P* < 0.01) results are given in bold

## Discussion

### The role of climate, soils-geomorphology and dispersal filters as driver of phylogenetic and taxonomic turnover

Climate was a very strong predictor of taxonomic and phylogenetic turnover at broader scales (Table 3B), in accordance with H1 and our results are also in agreement with recent evidence suggesting that climate is an important driver of tree species distribution and changes in species composition at regional scales (Baldeck et al. 2016; Esquivel-Muelbert et al. 2016, 2017; Neves et al. 2020). However, we also found a strong correlation between climatic distances and taxonomic turnover at finer spatial scales, similar to previous studies that demonstrated a strong effect of variation in drought sensitivity on tropical tree species distribution at local scales (Engelbrecht et al. 2007). Likewise, our results conflict with our expectation of high taxonomic and high phylogenetic turnover mediated by climatic distances (limited climatic beta niche evolution). We found patterns of low phylogenetic turnover and high taxonomic turnover with respect to climatic distances, contrasting with previous studies that reported high phylogenetic and high taxonomic turnover with respect to climatic variables (Hardy et al. 2012; Eiserhardt et al. 2013). High taxonomic but low phylogenetic turnover with climatic differences may be the result of recent climatic niche evolution, determining phylogenetic clustering towards the tips of the regional phylogenetic tree. This means that clusters of closely related species occupy contrasting climatic regions in the Ecuadorian Amazon. Our results also suggest that even small climatic fluctuations at local and landscape scales can contribute to this pattern (Table 3). It is important to note that a strong west–east gradient in precipitation and temperature in Ecuadorian Amazon (Figure S3, Mapa de Vegetación del Ecuador 2013) might promote lineages adapted to wet conditions to dominate forests to the west of basin while lineages recently adapted to drier conditions should be more dominant toward the east. Nonetheless, further studies should test this hypothesis focusing on testing the role of Neogene Quaternary-Miocene climatic fluctuations produced by the Andean uplift by explicitly testing for climatic niche evolution via ancestral climatic reconstructions.

Regarding H2, the hypothesis of geomorphological and edaphic control on Amazonian tree communities, we found mixed results. Although we found evidence for high taxonomic turnover with variation in soils and geomorphology at all spatial scales the effect of this relationship was not significant to explain patterns of phylogenetic turnover at broad spatial scales (Table 3A). Likewise, our results are not consistent with a scenario of high taxonomic and high phylogenetic turnover mediated by edaphic differences. Moreover, in contrast to H2 our results suggest that soil variation related to geomorphology is not a significant predictor of phylogenetic turnover at broad spatial scales (Table 3B). We also found that the spatially structured soil component was also not significantly associated with phylogenetic turnover at broad scales. However, when considered on their own, geomorphology and soils remain significant predictors of taxonomic and phylogenetic turnover at broad spatial scales. It is important to note that geomorphological attributes can be also related to unmeasured hydrological variables that we did not consider in our study. Geomorphological variables from DEM and related to relative elevation and topographic wetness above stream may define hydrological landscape factors determining changes in species composition in response to differences in flooding regimes (Baldeck et al. 2016). In our study, we were not able to include information on hydrology thus we acknowledge that the influence of some geomorphological variables might be underestimated.

Previous studies have concluded that geomorphology and their associated soil characteristics are the predominant factor in explaining taxonomic turnover in Amazonian plant communities at landscape and regional scales (Phillips et al. 2003; Higgins et al. 2011; Honorio Coronado et al. 2015; Tuomisto 2019). Such studies have pointed to a dichotomy between older Miocene-originated geological formations (e.g. Pebas Formation in Peru or the analogous Solimões Formation in Brazil), associated with rich nutrients, and the younger Pleiostecene–Pliocene-originated geological formations (e.g. the Iça Formation in Brazil, the Nauta Formation in Peru) associated with low-nutrient sediments (Sombroek 2000; Higgins et al. 2011; Honorio Coronado et al. 2015; Tuomisto et al. 2016, 2019). While this dichotomy might be useful to explain the patterns seen in those studies, we believe the geological history of western Amazonia and particularly in Ecuadorian Amazon is far more complex, making it difficult to find a clear relationship between geology and changes in tree species composition across the region (Figure S2).

**Fig. 4:**
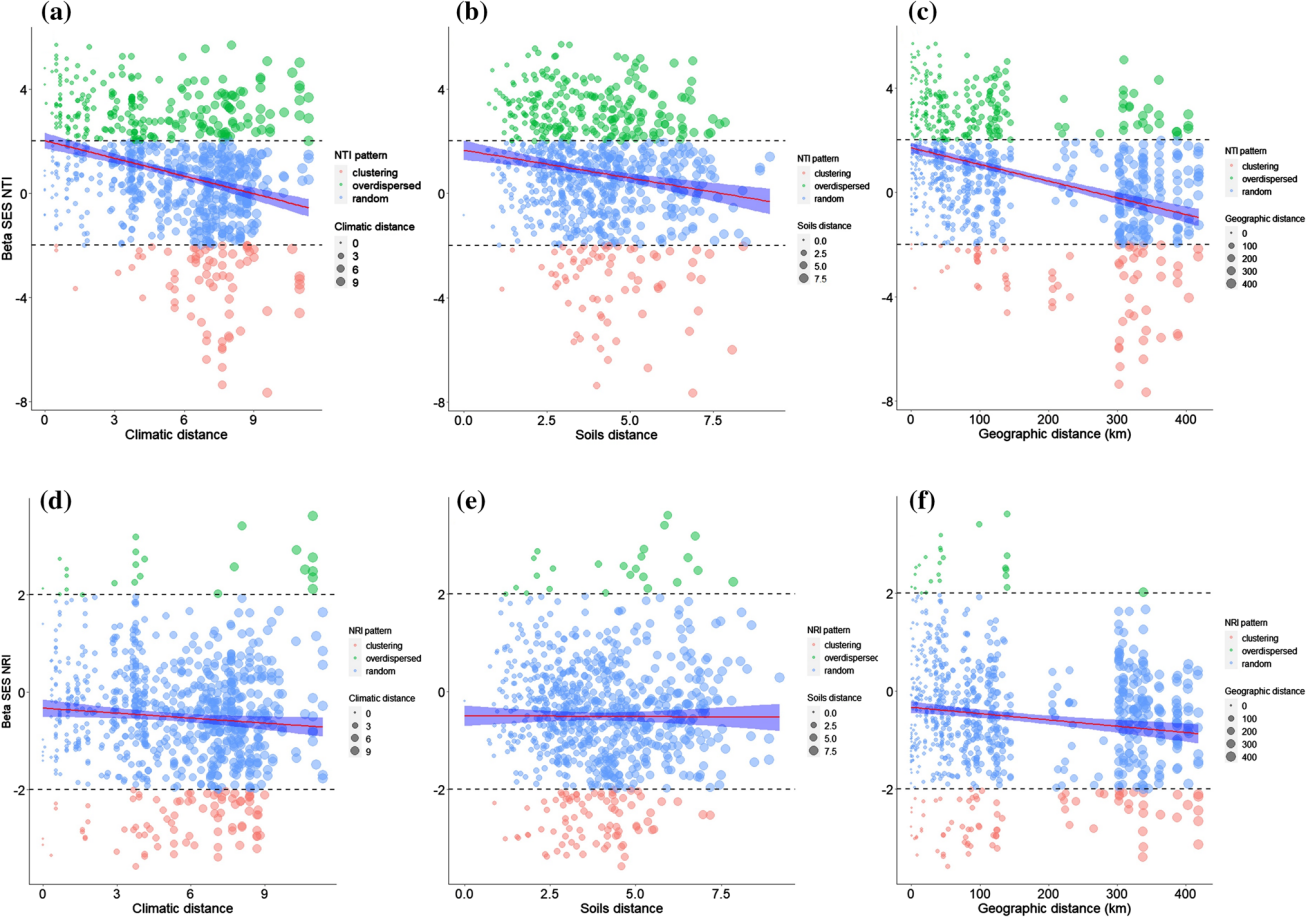
The inverse of the standardized effect size of phylobetadiversity (BetaNRI and BetaNTI) versus **a**, **d** climatic, **b**, **e** soils and **c**, **f** geographic distance separating tree communities in Ecuadorian Amazon. The red solid line indicates best fit from linear regression of BetaNRI and BetaNTI vs climatic, soils and geographic distance. The dashed lines represent the expectation under a null model of random shuffling of taxa across the tips of the regional phylogenetic tree, blue shaded region indicates 95% confidence intervals around the null expectation (mean ± 1.96SD). Positive values indicate phylogenetic evenness, negative values indicate phylogenetic clustering. Pairs of communities outside the 95% confidence interval are significantly more clustered or even with respect to one another than expected by chance; dot sizes are weighted by the increase in climatic, edaphic and geographic distances. Environmental variables were first scaled and then Euclidean distances calculated. Geographic distances are based on Euclidean distances alone

In agreement with H3 we found that the effect of fine scale edaphic variation was a strong predictor of high species but low phylogenetic turnover. Two main ideas may help to explain the patterns we found. First of all, a geologically and edaphically complex system like the western Amazon may promote parapatric or “mosaic sympatric” speciation (Gentry 1986; Mallet 2008). Divergent natural selection on the boundaries of soils habitats with strong differences should trigger adaptations to one or the other habitat, leading to stronger taxonomic turnover than lineage turnover in tree communities between soil types associated with a particular geological formation (Fine and Kembel 2011). Thus, phylogenetic clustering among pairs of tree communities towards the tips of the regional phylogenetic tree might result from recent divergence mediated by habitat specialization to different soils (Fig. 4). On the other hand, we would expect higher values of phylogenetic turnover if trait conservatism for the soil niche axis is prevalent in the tree community and early divergent lineages show extensive turnover between soil types (H2).

Secondly, while we agree that soils have a strong influence at local and landscape scales on patterns of tree taxonomic turnover (H3), we posit that this effect might not be as strong for Amazonian tree communities at regional scales and large taxonomic scales. Evidence for niche lability related to soil conditions has been found in tropical tree communities, meaning that niche conservatism related to habitat specialization might not be a ubiquitous pattern (Baldeck et al. 2013; Fine et al. 2006; Fine and Baraloto 2016). This has strong implications in detecting patterns of both lineage and taxonomic turnover, because if the niche axis related to soils is labile in most Amazonian tree lineages this might produce low phylogenetic turnover across soil gradients (Anacker and Harrison 2012).

**Fig. 5:**
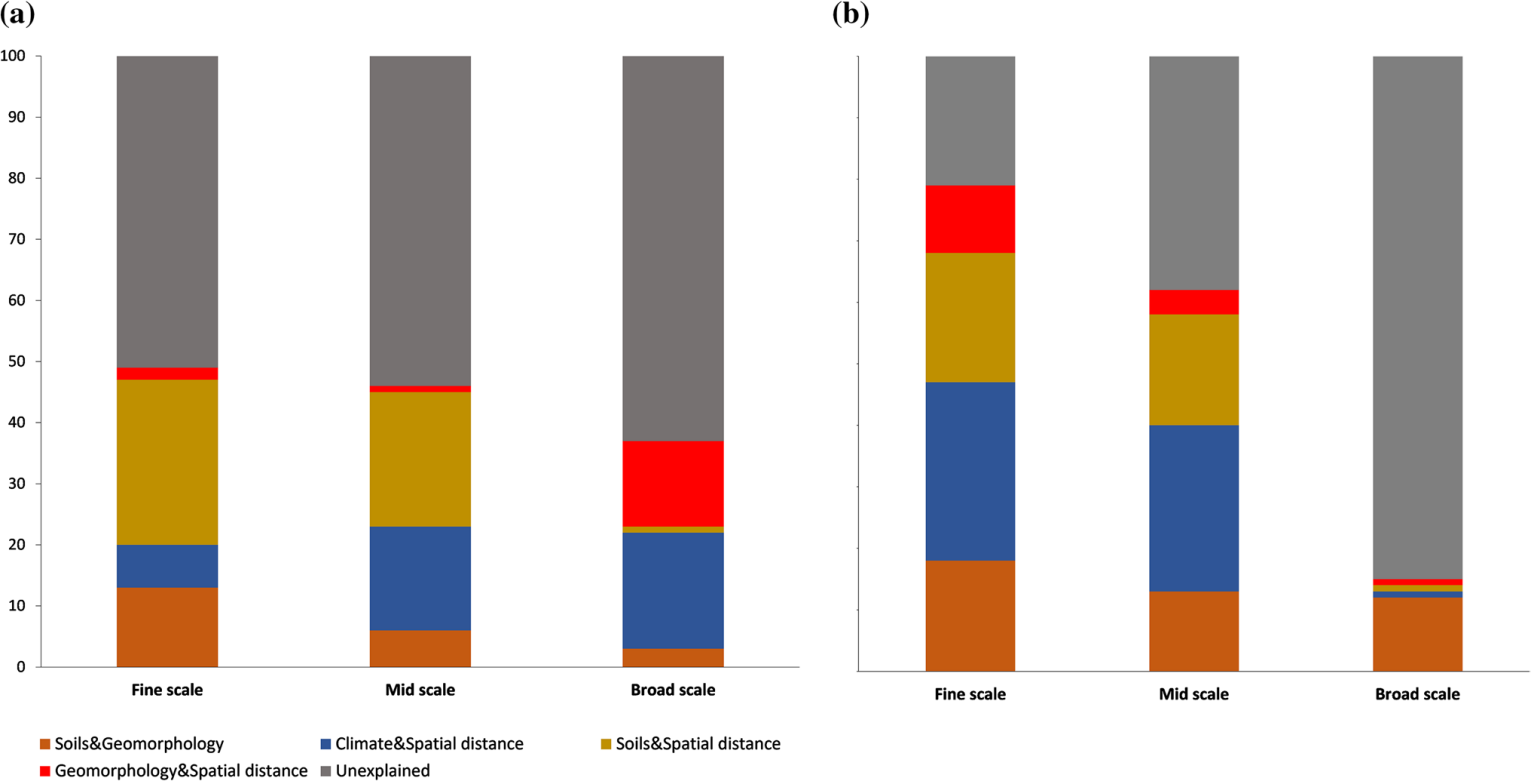
Variation partitioning analysis using redundancy analysis (RDA) explaining the fraction of variation in **a** taxonomic turnover and **b** phylogenetic turnover in Ecuadorian Amazon tree communities explained by climate, geomorphology, soils and pure spatial variables (geography); the fractions represent the combined contribution of geomorphology-soils, climate-spatial distance, soils-spatial distances, geomorphology-spatial distances and the fraction of variation unexplained by the combination of all variables. Broad, mid and fine spatial scales are defined on the basis of PCNMs eigenvectors analysis

In accordance with H4, our results suggest that geographic distance as a proxy of dispersal limitation has as strong an effect as climate on patterns of taxonomic turnover. The influence of geographic distances is stronger at broader spatial scales suggesting that dispersal limitation acts as a strong filter for tree local community assembly. The importance of dispersal limitation as a driver of changes in species composition of Amazonian forests has been previously demonstrated in many studies (Hubbell 2001; Condit et al. 2002; Terborgh et al. 2011; Cárdenas et al. 2017). Pure spatial distances may be used as a proxy for “dispersal barriers” but also could serve as a proxy for “time for dispersal” processes, including differences in the historical biogeography of Amazonian tree lineages. Our results suggest that dispersal limitation drives taxonomic turnover at distances equal to or over 50 km suggesting that potential dispersal barriers act more strongly at mid or large spatial scales. However, because time for dispersal implies the ability for species to disperse across uniform suitable environments, it is hard to disentangle its effects from the effects that potential barriers might impose for dispersal limitation. Thus, additional analyses that incorporate barrier distances are needed to fully understand the role of dispersal limitation (Eisehardt et al. 2013).

We show that dispersal limitation likely operates over evolutionary time scales because this mechanism may require some level of niche conservatism to act as an important filter producing a correlation between phylogenetic turnover and geographic distances (H4) (Wiens and Graham 2005; Eiserhardt et al. 2013). Additionally, our finding that a large proportion of the variation in phylogenetic turnover is largely explained by the spatially structured component of the environment supports the idea that spatial distance might act together with environmental filters as drivers of the patterns we described. In fact, the spatially structured climatic component was significantly associated with phylogenetic turnover at mid spatial scales and highly significant at broad scales (17% and 19% of explained variation, respectively). Phylogenetic turnover not only is related to the spatial variability in lineage composition but should also be related to variability in the set of traits for subsets of the regional species pool (Weinstein et al. 2014). Unsuitable regions defined by climatic niches may act as barriers for dispersal precluding some lineages without physiological traits that allow them to colonizing and establish themselves in areas that experienced divergent climates. Therefore, limited climatic niche evolution may act together with dispersal limitation to determine the patterns of phylogenetic turnover we observed. Our results are at odds with a recent study that proposed a limited effect of dispersal limitation in the assembly of Amazonian tree communities on an evolutionary scale (Dexter et al. 2017). While we agree that for some tree lineages there is evidence that on evolutionary timescales, the metacommunity for any regional or local tree community in the Amazon could be the entire Amazon basin, we argue that evolutionary constraints on tree dispersal modes acts as a mechanism driving changes in phylogenetic composition across space. In fact, our analysis of both terminal and basal phylogenetic beta diversity (beta NTI beta NRI) confirm this hypothesis. Phylogenetic clustering was strongly associated with geographic distances towards the tips of the tree and tree wide showing that potential dispersal limitation has strong phylogenetic signal.

## Conclusions

We posit that the combined effect of geomorphology and soils are not the main driver of phylogenetic turnover at large spatial scales but instead are more important for taxonomic turnover at finer spatial scales, at least for tree communities in the Ecuadorian Amazon. On the other hand, climate and dispersal limitation drives phylogenetic turnover patterns at regional scales by filtering out lineages with potential physiological constraints to occupy unsuitable climates. Thus, we suggest that climate, rather than geomorphology or soils, operates as the main driver for the clade composition of biogeographic regions by influencing speciation and extinction processes related to physiological constraints (Lessard et al. 2011; Mittelbach and Schemske 2015). Certainly, geomorphology, geology and soils play an important role in Amazonian plant species composition and its influence may be fundamental for tree community assembly at local scales (Vormisto et al. 2004; Higgins et al. 2011; Tuomisto et al. 2016, 2019). However, the dichotomization of old vs. young geological formations, and their underlying soil differences that lead to abrupt shifts in tree species composition at biogeographic scales, should not be considered as the main factor in understanding the assembly of Amazonian tree communities at large scales.

Our results are also connected with recent evidence that suggests a fundamental role of climate-induced changes in Amazonian tree species composition at short temporal scales. A slow shift to more dry-affiliated taxa is underway across the Amazon basin and may be the result of climate change drivers (Esquivel-Muelbert et al. 2018). This effect is also evident in regions such as Western Amazon where a decrease in abundance of wet-affiliated taxa is changing functional and species composition at broad scales (Esquivel-Muelbert et al. 2018). Thus, we argue that on the face of climate change it is imperative to understand the synergistic effects of environmental drivers on phylogenetic, functional and taxonomic composition of Amazon forests.

## Supplementary Information

Below is the link to the electronic supplementary material.

Supplementary file1 (XLSX 21 kb)

Supplementary file2 (DOCX 8498 kb)

## Data Availability

The datasets GENERATED/ANALYZED for this study can be found in the Dryad repository. https://datadryad.org/stash/share/8y1skZUwQuluqaFXrAI0SZaw2O1fhYIpO1nMkTYH5x8.
