# Supplementary material for: The contribution of environmental and dispersal filters on phylogenetic and taxonomic beta diversity patterns in Amazonian tree communities
Source: Oecologia. 2021 Jul 29;196(4):1119–37. doi: 10.1007/s00442-021-04981-0 (PMC8367926; doi:10.1007/s00442-021-04981-0)
Supplement: Supplementary file 1 — Supplementary file1 (XLSX 21 kb) [file 442_2021_4981_MOESM1_ESM.docx]

Supplementary Material

**The contribution of environmental and dispersal filters on beta diversity patterns in Amazonian tree communities**

Juan Ernesto Guevara Andino, Nigel C.A. Pitman, Hans ter Steege, Manuel Peralvo, Carlos Cerón, Paul V.A. Fine

**Tree sampling in Ecuadorian Amazon**

The 40 one hectare plot work used in this study are part of the Amazon Tree diversity Network and have been established by the lead author and co-authors in the past 20 years representing an unprecedented data set. In the past four years, we established 15 one-hectare plots in both terra firme and white sand forests in the Ecuadorian Amazon. We established 8 plots on alluvial terraces of the Aguarico River towards the north of the Ecuadorian Amazon; the age of these units ranges from Pliocene to Pleistocene origin (<http://www.geoinvestigacion.gob..ec>, Laraque *et al.*2009) The landscape is mostly characterized by large areas that correspond to Pleistocene alluvial terraces that occasionally suffer flooding events. These geomorphological units are interrupted only by high terraces with flat surfaces that have not suffered erosion of their surfaces (Saunders 2008; Wesselingh *et al.* 2006). Two additional plots were established in old alluvial terraces of Napo River, these units as well as the units located in the Aguarico River are high terraces that presumably constitute old flood plains of the previously mentioned rivers (Saunders 2008). Ten plots were established in the Pastaza megafan which is a massive alluvial deposit located in the southwestern Ecuadorian Amazon, evidence suggests that the modern megafan complex dates from the Pliocene-Pleistocene and recent alluvial processes have occurred between the last 180 000-30 000 yrs. (Rasanen *et al.* 1995; Bernal *et al.* 2011). Five plots were established in areas belonging to plateaus originated during the Cretaceous period; these geomorphological units are located in the lowest part of Cordillera del Condor below 500 m.

The remaining 25 one hectare plots were established in the Yasuní national Park and the Tigre-Corrientes watershed. The landscape in both areas is characterized by the predominance of geomorphological units such as highly dissected hills occasionally interrupted by valleys (Pitman 2000). The landscape is dominated by Curaray and Chambira formations from Miocene and Mio-Pliocene origin respectively and soils are characterized by higher nutrients content (Pitman *et al.* 2008).

**Geomorphological variables and proximity analysis**

Four geographic variables (hierarchical slope position, slope, dem and landsat) were used in the analysis describing the geomorphology and land cover features in the vicinity of the forest plots. Digital terrain elevation data for the Ecuadorian Amazon was obtained from the Shuttle Radar Topography Mission (SRTM) distributed by the USGS through the Earth Explorer platform (<https://earthexplorer.usgs.gov/>). The SRTM dataset has worldwide coverage of void filled elevation data at a resolution of 1 arc-second (30 meters). Topographic slope in degrees was calculated from the elevation data using the Spatial Analyst extension in ArcGIS 10.3 software from ESRI (Environmental Systems Resource Institute).

Hierarchical Slope Position identifies topographic exposure (ridge, slope, valley bottom, etc) by applying moving windows with increasing radii to a digital elevation model (DEM) (Murphy et al. 2010). The exposure is a ridge if the elevation of the center cell in the window is higher than the average of the cells in the window. The opposite case corresponds to a valley bottom or toe slope. Hierarchical integration is done by starting with exposure values for the largest (user defined) window and adding values from smaller windows if their absolute standardized values exceed the values of the larger scale map. The variable was calculated using the Geomorphometry and Gradient Metrics (version 2.0) for ArcGIS (Evans et al. 2014) using windows of radii between 2 and 10 pixels with increments of two pixels.

Slope position was measured by subtracting the Slope Position average neighbor values from a focal value. Positive values indicate that the central point is located higher than its average surroundings, while negative indicates a position lower than the average. The range of the metric depends not only on differences but also on the defined neighborhood. This metric is also referred to as Topographic Position Index (TPI) (Guisan et al. 1999).

Digital elevation models (DEM) measures the bare-earth surface based on raster grids of the elevation between two or more points. We obtained data for Ecuadorian Amazon from the SRTM 90-meter resolution Digital Elevation Model developed by the NASA. We concatenated the different mosaics of topography in ArcMap 10.5.1 using the Spatial Statistics tool.

Land cover information was obtained from a mosaic of Landsat images for the period 2010-2014 that had been created for the Ministry of the Environment of Ecuador. The mosaic was was created using the approach described in Hansen et al. (2013) that includes (i) image resampling, (ii) conversion of raw digital values (DN) to top of atmosphere (TOA) reflectance, (iii) cloud/shadow/water screening and quality assessment (QA), and (iv) image normalization. A principal components analysis was performed using the three RGB bands of the mosaic and the first component, that explained 96.4% of the variance, was used for further analysis.

For each plot, a set of circular buffers with areas of 0.5 km^2^, 5 km^2^ and 50-100 km^2^. Descriptive statistics were calculated for each variable at each scale defined by the buffer areas using zonal statistics tools in ArcGIS.

**References**

Bernal, C., Christophoul, F., Darrozes, J., Soula, J.C., Baby, P. & Burgos, J. (2010). Late Glacial and Holocene avulsions of the Rio Pastaza Megafan (Ecuador–Peru): frequency and controlling factors. *International Journal of Earth Science,* 100, 1759–1782.

Evans J.S., Oakleaf J., Cushman S.A. & Theobald D. (2014). An ArcGIS Toolbox for Surface Gradient and Geomorphometric Modeling, version 2.0-0. Available: http://evansmurphy.wix.com/evansspatial. Accessed: 12/05/2016.

Guisan, A., Weiss, S.B. & Weiss, A.D. (1999) GLM versus CCA spatial modeling of plant species distribution. *Plant Ecology* 143:107–122.

Hansen, M. C., P. V. Potapov, R. Moore, M. Hancher, S. A. Turubanova, A. Tyukavina, D. Thau, S. V. Stehman, S. J. Goetz, T. R. Loveland, A. Kommareddy, A. Egorov, L. Chini, C. O. Justice, and Townshend, J.R.G. (2013). High-Resolution Global Maps of 21st-Century Forest Cover Change. *Science* 342 (6160):850-853.

Laraque, Bernal, C., Bourrel, L., Darrozes, J., Christophoul, F., Armijos, E., Fraizy, P., Pombosa, R. & Guyot, J. L. (2009). Sediment budget of the Napo River, Amazon basin, Ecuador and Peru. *Hydrological Processes,* 23, 3509–3524.

Murphy, M.A., Evans, J.S. & Storfer, A. (2010). Quantifying Bufo boreas connectivity in Yellowstone National Park with landscape genetics. *Ecology* 91 (1):252-261.

Rasanen, M.E., Salo, J.S., Jungner, H. & Pittman, L.R. (1990). Evolution of the western Amazonian lowland relief: impact of Andean foreland dynamics. *Terra Nova*, 2, 320–332.

Saunders, T. J. (2008). Geología, hidrología y suelos: procesos y propiedades del paisaje. Ecuador-Perú: Cuyabeno-Güeppí. Pp 66-75 in Rapid Biological and Social Inventories. Alverson, W. S., C. Vriesendorp, Á. del Campo, D. K. Moskovits, D. F. Stotz, M. García D. and L. A. Borbor L., eds. Report 20. The Field Museum, Chicago.

Wessenlingh, F. P., Hoorn, M. C., Guerrero, J., Räsänen, M., Romero Pitmann, L. & Salo, J. (2006)**.** The stratigraphy and regional structure of Miocene deposits in western Amazonia (Peru, Colombia and Brazil), with implications for late Neogene landscape evolution. *Scripta Geologica* **133**:291 – 322.

Table S1. Eigenvector functions for an 80 one-hectare plot network in Ecuadorian Amazon. This data set includes the 41 plots used in this study; asterisks represent statistical significance at 5% after a forward selection procedure implemented in the packfor package in the R statistical software.

|  | PCNM1 * | PCNM2 * | PCNM10* | PCNM11* | PCNM24 | PCNM28 |
| --- | --- | --- | --- | --- | --- | --- |
| ALTA_FLOR | -0.1215 | 0.1615 | -0.0020 | -0.0355 | 0.1400 | 0.0096 |
| ALTA_FLOR2 | -0.1215 | 0.1613 | -0.0021 | -0.0355 | 0.1190 | 0.0082 |
| BALSAURA | -0.0796 | -0.0494 | 0.6032 | 0.0075 | 0.0001 | 0.0000 |
| BATABURO | -0.0273 | -0.0283 | -0.0206 | -0.0568 | 0.0005 | 0.0000 |
| BOG_01 | 0.1337 | -0.0088 | -0.0003 | 0.0350 | 0.0168 | 0.0368 |
| BOG_02 | 0.1338 | -0.0087 | -0.0004 | 0.0349 | 0.0443 | 0.0290 |
| BOG_03 | 0.1338 | -0.0087 | -0.0004 | 0.0349 | 0.0437 | 0.0263 |
| BOG_04 | 0.1337 | -0.0088 | -0.0003 | 0.0349 | 0.0155 | 0.0314 |
| BOG_05 | 0.1337 | -0.0088 | -0.0003 | 0.0350 | 0.0161 | 0.0341 |
| BOG_06 | 0.1349 | -0.0034 | -0.0022 | -0.1657 | -0.0740 | 0.0455 |
| BUFEO | -0.0860 | -0.0578 | 0.1166 | 0.0212 | -0.0007 | -0.0008 |
| CAN_01 | 0.1353 | -0.0064 | 0.0097 | 0.0639 | 0.0120 | -0.2710 |
| CEIBA | 0.1351 | -0.0032 | -0.0023 | -0.1640 | 0.0120 | 0.0516 |
| CHIWIAS | -0.1196 | -0.2164 | 0.0644 | 0.0039 | -0.0021 | 0.0031 |
| CHUCULA | -0.1273 | 0.1944 | 0.0140 | 0.1561 | 0.0604 | -0.0025 |
| CONAMBO | -0.0682 | -0.0313 | 0.0119 | 0.0009 | -0.0001 | 0.0000 |
| CYB_LG_1 | -0.0162 | 0.0192 | -0.0014 | -0.1336 | -0.0002 | 0.5020 |
| CYB_LG_2 | -0.0162 | 0.0192 | -0.0014 | -0.1336 | 0.0002 | -0.5020 |
| DICAM_01 | 0.1241 | 0.0350 | 0.0012 | 0.0501 | -0.0484 | 0.0210 |
| DICAM_02 | 0.1241 | 0.0350 | 0.0012 | 0.0501 | -0.0479 | 0.0186 |
| DICARO_01 | 0.1239 | 0.0351 | 0.0012 | 0.0510 | -0.0212 | 0.0466 |
| DICARO_02 | 0.1238 | 0.0351 | 0.0012 | 0.0510 | -0.0206 | 0.0441 |
| DICARO_03 | 0.1239 | 0.0351 | 0.0012 | 0.0510 | -0.0220 | 0.0502 |
| EC_JUY | -0.0678 | -0.0309 | 0.0101 | -0.0008 | 0.0000 | 0.0000 |
| GUEPI | -0.1019 | 0.1229 | -0.0101 | -0.2294 | -0.0061 | 0.0075 |
| GUEPPI | -0.1019 | 0.1229 | -0.0101 | -0.2294 | 0.0016 | -0.0075 |
| HERRADURA | 0.1351 | -0.0032 | -0.0023 | -0.1638 | 0.0297 | 0.0752 |
| HOATZIN | -0.1272 | 0.1943 | 0.0141 | 0.1565 | 0.1680 | -0.0028 |
| JAGUAR | 0.1350 | -0.0033 | -0.0023 | -0.1642 | -0.0059 | 0.0314 |
| JAS_02 | -0.0747 | -0.0357 | -0.1915 | 0.0326 | 0.0007 | -0.0001 |
| JAS_03 | -0.0747 | -0.0357 | -0.1912 | 0.0326 | 0.0007 | 0.0006 |
| JAS_04 | -0.0747 | -0.0357 | -0.1915 | 0.0326 | 0.0007 | -0.0003 |
| JAS_05 | -0.0749 | -0.0358 | -0.1917 | 0.0329 | 0.0009 | -0.0004 |
| KAMBA_01 | -0.1076 | -0.1881 | -0.0267 | 0.0059 | 0.0001 | -0.0005 |
| KAMBA_02 | -0.1076 | -0.1881 | -0.0267 | 0.0059 | 0.0001 | -0.0001 |
| KAMPA | -0.1076 | -0.1882 | -0.0266 | 0.0059 | 0.0002 | 0.0017 |
| KAPAWI1 | -0.0849 | -0.0691 | -0.2739 | 0.0130 | 0.0006 | 0.0008 |
| KAPUT_01 | -0.1076 | -0.1882 | -0.0266 | 0.0059 | 0.0002 | 0.0021 |
| KAPUT_02 | -0.1076 | -0.1881 | -0.0267 | 0.0059 | 0.0001 | 0.0004 |
| KAPUT_03 | -0.1076 | -0.1881 | -0.0267 | 0.0059 | -0.0001 | -0.0027 |
| KM21 | 0.1346 | -0.0067 | 0.0103 | 0.0653 | -0.0249 | 0.0332 |
| KURINTZA | -0.0817 | -0.0479 | -0.0471 | 0.0180 | 0.0027 | 0.0010 |
| PAKINTZA | -0.0972 | -0.1199 | 0.3640 | 0.0061 | 0.0000 | 0.0000 |
| PANTANO | 0.1349 | -0.0033 | -0.0023 | -0.1642 | -0.0235 | -0.0087 |
| PARCHE | -0.1359 | 0.2144 | 0.0046 | -0.0692 | -0.2670 | 0.0026 |
| PAYAMINO | -0.0570 | -0.0276 | -0.0094 | 0.1573 | 0.0005 | 0.0001 |
| PILIMOSCA | -0.1360 | 0.2144 | 0.0046 | -0.0693 | -0.2210 | 0.0024 |
| PIR_01 | 0.1354 | -0.0063 | 0.0096 | 0.0635 | 0.0286 | -0.3300 |
| SALADERO | 0.1301 | -0.0033 | -0.0231 | -0.0482 | -0.1330 | -0.1560 |
| SANGUIJELA | 0.1350 | -0.0033 | -0.0023 | -0.1643 | -0.0097 | 0.0386 |
| SAWASTIAN | -0.0936 | -0.1158 | -0.2041 | -0.0012 | 0.0000 | 0.0000 |
| SEWAYA | 0.1245 | 0.0316 | 0.0125 | -0.1086 | 0.0078 | -0.3060 |
| SHI_01 | 0.1342 | -0.0067 | 0.0105 | 0.0658 | -0.0207 | 0.1880 |
| SHI_02 | 0.1342 | -0.0067 | 0.0106 | 0.0658 | -0.0201 | 0.1910 |
| SHI_03 | 0.1342 | -0.0067 | 0.0106 | 0.0659 | -0.0195 | 0.1950 |
| SHIRAM_E | -0.1200 | -0.2180 | 0.0611 | 0.0042 | 0.0013 | -0.0017 |
| SHR_01 | -0.0652 | -0.0279 | 0.0589 | -0.1355 | 0.0000 | 0.0000 |
| SHUSHUFINDI | 0.0589 | -0.0312 | 0.0225 | -0.2949 | 0.0007 | 0.0000 |
| STA_TERESITA | -0.1244 | 0.1780 | 0.0068 | 0.1048 | 0.0000 | -0.0005 |
| TARANGARO | -0.0808 | -0.0420 | 0.1434 | -0.0325 | 0.0015 | 0.0001 |
| TIGRILLO | 0.1351 | -0.0031 | -0.0023 | -0.1631 | 0.0636 | 0.0809 |
| TINKIAS | -0.0765 | -0.0484 | -0.4510 | 0.0136 | -0.0007 | -0.0008 |
| TIP_01 | 0.1322 | -0.0003 | -0.0131 | -0.0148 | 0.1890 | -0.0728 |
| TIP_02 | 0.1259 | 0.0315 | 0.0023 | 0.1789 | -0.1210 | -0.0092 |
| TIP_03 | 0.1322 | -0.0003 | -0.0131 | -0.0148 | 0.1890 | -0.0728 |
| TIP_04 | 0.1346 | -0.0067 | 0.0101 | 0.0646 | -0.0876 | -0.1750 |
| TIP_05 | 0.1259 | 0.0315 | 0.0023 | 0.1789 | -0.1200 | -0.0061 |
| TSUIRIM | -0.1115 | -0.1979 | -0.0165 | 0.0019 | -0.0007 | -0.0008 |
| TUKUP | -0.1198 | -0.2174 | 0.0602 | 0.0041 | 0.0028 | -0.0014 |
| VILLANO | -0.0781 | -0.0398 | 0.1124 | 0.0344 | -0.0015 | -0.0001 |
| WASURAK | -0.0953 | -0.1292 | 0.0115 | -0.0072 | 0.0000 | 0.0000 |
| YARINA | -0.1359 | 0.2143 | 0.0046 | -0.0694 | -0.2520 | 0.0023 |
| YAS_JC | -0.1209 | 0.1598 | -0.0024 | -0.0352 | -0.4910 | -0.0123 |
| YASUNI_JG | 0.1232 | 0.0354 | 0.0012 | 0.0528 | 0.0439 | 0.0588 |
| YUTURI | 0.1224 | 0.0356 | 0.0012 | 0.0545 | 0.1090 | 0.0800 |
| ZANCUDO | -0.1363 | 0.2156 | 0.0048 | -0.0671 | 0.1830 | -0.0015 |
| ZANCUDO_02 | -0.1363 | 0.2157 | 0.0048 | -0.0678 | 0.5580 | -0.0056 |
| ZOJECHOE | -0.0604 | 0.1870 | 0.0142 | 0.1026 | -0.0005 | 0.0000 |
| ZOJECHOE_02 | -0.0644 | 0.1986 | 0.0188 | 0.2859 | -0.0025 | 0.0000 |
|  |  |  |  |  |  |  |

Table S2. Results for axis 1 and 2 of a non-metric multidimensional analysis based on taxonomic (1- Sorenson) and phylogenetic turnover (1-Phylosorenson) for an 80 one-hectare plot network in Ecuadorian Amazon. This data set includes the 41 plots used in this study. The age of the geological formation is also shown. Both the type of formation and the age of each geological formation were obtained from the geological map developed by the Instituto de Investigaciones Geológicas Mineras Metalúrgicas of Ecuador.

|  | **Geology** | **Latitude** | **Longitude** | **No of species** | **Individuals** | **Forest** | **NMDS1 phylogeny** | **NMDS1 taxonomy** |
| --- | --- | --- | --- | --- | --- | --- | --- | --- |
| ALTA_FLOR | Pleistocene | -0.889 | -75.458 | 233 | 476 | TF | -0.12008 | -0.08431 |
| ALTA_FLOR2 | Pleistocene | -0.896 | -75.462 | 131 | 313 | TF | -0.19358 | 0.035613 |
| BALSAURA | Pliocene | -1.9347 | -77.281 | 119 | 566 | TF | -0.06327 | 0.027695 |
| BATABURO | Miocene | -1.2 | -76.7167 | 197 | 452 | TF | -0.06912 | -0.07153 |
| BOG_01 | Miocene | -0.6982 | -76.4833 | 206 | 507 | TF | 0.128545 | -0.1782 |
| BOG_02 | Miocene | -0.6991 | -76.4667 | 212 | 513 | TF | 0.164709 | -0.19023 |
| BOG_03 | Miocene | -0.7 | -76.4667 | 196 | 501 | TF | 0.133953 | -0.16745 |
| BOG_04 | Miocene | -0.7 | -76.4833 | 216 | 582 | TF | 0.11137 | -0.18318 |
| BOG_05 | Miocene | -0.6991 | -76.4833 | 199 | 504 | TF | 0.2044 | -0.20061 |
| BOG_06 | Miocene | -0.66667 | -76.4333 | 194 | 576 | TF | 0.002939 | -0.09337 |
| BUFEO | Mio-Pliocene | -2.19554 | -76.771 | 102 | 338 | TF | -0.06144 | 0.072124 |
| CAN_01 | Miocene | -0.63333 | -76.4667 | 254 | 687 | TF | 0.118823 | -0.22063 |
| CEIBA | Mio-Pliocene | -0.66478 | -76.3839 | 112 | 435 | VA | 0.140565 | 0.024499 |
| CHIWIAS | Pleistocene | -2.66556 | -77.4983 | 128 | 398 | TF | 0.122359 | -0.1022 |
| CHUCULA | Pleistocene | -0.63417 | -75.2358 | 90 | 480 | VA | 0.084553 | 0.159236 |
| CONAMBO | Miocene | -1.82464 | -76.6975 | 173 | 715 | TF | -0.02605 | -0.0786 |
| CYB_LG_1 | Pleistocene | -0.01677 | -76.1833 | 140 | 428 | TF | -0.12913 | 0.045323 |
| CYB_LG_2 | Pleistocene | -0.01577 | -76.1833 | 146 | 545 | TF | -0.2509 | 0.122612 |
| DICAM_01 | Mio-Pliocene | -0.56667 | -76.1333 | 32 | 164 | SW | 0.225514 | 0.241755 |
| DICAM_02 | Mio-Pliocene | -0.56577 | -76.1333 | 66 | 382 | SW | 0.212575 | 0.159172 |
| DICARO_01 | Mio-Pliocene | -0.56667 | -76.1167 | 208 | 458 | VA | 0.169275 | -0.19325 |
| DICARO_02 | Mio-Pliocene | -0.56577 | -76.1167 | 205 | 505 | VA | 0.146779 | -0.14619 |
| DICARO_03 | Miocene | -0.568 | -76.1167 | 49 | 760 | SW | -0.04817 | 0.241314 |
| EC_JUY | Miocene | -2.13316 | -76.2011 | 163 | 442 | TF | -0.13921 | 0.005987 |
| GUEPI | Miocene | -0.11 | -75.524 | 154 | 463 | TF | -0.12261 | 0.063642 |
| GUEPPI | Miocene | -0.11 | -75.525 | 59 | 249 | IG | 0.043095 | 0.205238 |
| HERRADURA | Mio-Pliocene | -0.658 | -76.3763 | 65 | 307 | VA | 0.168938 | 0.108425 |
| HOATZIN | Miocene | -0.6133 | -75.2333 | 98 | 409 | IG | 0.195769 | 0.026202 |
| JAGUAR | Mio-Pliocene | -0.67066 | -76.392 | 114 | 331 | VA | 0.144577 | -0.00943 |
| JAS_02 | Pleistocene | -1.06667 | -77.6147 | 180 | 537 | TF | 0.050506 | -0.10715 |
| JAS_03 | Miocene | -1.08 | -77.61 | 182 | 536 | TF | 0.03469 | -0.1468 |
| JAS_04 | Pleistocene | -1.06667 | -77.6167 | 136 | 534 | VA | 0.052848 | -0.07461 |
| JAS_05 | Pleistocene | -1.045 | -77.63333 | 140 | 501 | VA | 0.057598 | -0.07061 |
| JOY_SACH | Mio-Pliocene | -0.31417 | -76.8914 | 66 | 92 | TF | 0.244284 | -0.00143 |
| KAMBA_01 | Cretaceous | -3.01733 | -77.9167 | 72 | 519 | PZ | -0.50805 | 0.350913 |
| KAMBA_02 | Cretaceous | -3.01667 | -77.9167 | 139 | 516 | PZ | -0.16943 | 0.019177 |
| KAMPA | Cretaceous | -3.01722 | -77.9139 | 85 | 244 | PZ | -0.41536 | 0.333415 |
| KAPAWI1 | Pliocene | -2.51667 | -76.8333 | 141 | 355 | TF | -0.17936 | 0.042779 |
| KAPUT_01 | Cretaceous | -3.01632 | -77.9139 | 101 | 212 | PZ | -0.444 | 0.281056 |
| KAPUT_02 | Cretaceous | -3.01667 | -77.916 | 30 | 80 | PZ | -0.56894 | 0.504106 |
| KAPUT_03 | Cretaceous | -3.01667 | -77.92 | 78 | 488 | PZ | -0.42682 | 0.312063 |
| KM21 | Miocene | -0.55444 | -76.5206 | 241 | 706 | TF | 0.03064 | -0.17666 |
| KURINTZA | Mio-Pliocene | -2.06769 | -76.7524 | 128 | 366 | TF | -0.01282 | -0.03264 |
| PAKINTZA | Pleistocene | -2.35179 | -77.2848 | 104 | 364 | TF | -0.0696 | 0.112376 |
| PANTANO | Mio-Pliocene | -0.68287 | -76.3974 | 173 | 479 | VA | 0.124432 | -0.08693 |
| PARCHE | Miocene | -0.56972 | -75.2347 | 161 | 507 | TF | -0.01191 | -0.00546 |
| PAYAMINO | Pleistocene | -0.4667 | -77.1678 | 186 | 550 | TF | 0.008914 | -0.08821 |
| PILIMOSCA | Miocene | -0.56444 | -75.2381 | 49 | 334 | VA | -0.06955 | 0.29517 |
| PIR_01 | Miocene | -0.65 | -76.45 | 215 | 518 | TF | 0.061619 | -0.12719 |
| SALADERO | Miocene | -0.80833 | -76.3989 | 224 | 591 | TF | 0.056837 | -0.18482 |
| SANGUIJELA | Mio-Pliocene | -0.6684 | -76.3952 | 119 | 409 | VA | 0.136164 | 0.016184 |
| SAWASTIAN | Pleistocene | -2.63873 | -77.154 | 107 | 399 | TF | 0.186625 | -0.0683 |
| SEWAYA | Pleistocene | -0.29167 | -76.2723 | 162 | 385 | TF | -0.09112 | 0.028215 |
| SHI_01 | Miocene | -0.51667 | -76.5333 | 231 | 539 | TF | 0.085285 | -0.19919 |
| SHI_02 | Miocene | -0.51577 | -76.5333 | 234 | 596 | TF | 0.09617 | -0.19464 |
| SHI_03 | Miocene | -0.51487 | -76.5333 | 194 | 512 | TF | 0.058022 | -0.13627 |
| SHIRAM_E | Pleistocene | -2.74583 | -77.5606 | 94 | 244 | TF | -0.11551 | 0.091109 |
| SHR_01 | Pleistocene | -1.01667 | -76.9833 | 172 | 568 | TF | 0.077539 | -0.12508 |
| SHUSHUFINDI | Mio-Pliocene | -0.20667 | -76.6606 | 63 | 84 | TF | 0.227202 | 0.02891 |
| STA_TERESITA | Pleistocene | -0.853 | -75.464 | 150 | 449 | TF | -0.10715 | 0.038542 |
| TARANGARO | Pliocene | -1.47939 | -77.3294 | 101 | 151 | TF | 0.071226 | 0.019377 |
| TIGRILLO | Mio-Pliocene | -0.6575 | -76.3567 | 187 | 493 | TF | 0.057845 | -0.12541 |
| TINKIAS | Pliocene | -2.47939 | -76.7083 | 95 | 118 | TF | -0.17887 | 0.092065 |
| TIP_01 | Mio-Pliocene | -0.63333 | -76.2333 | 149 | 488 | VA | 0.192494 | -0.06039 |
| TIP_02 | Mio-Pliocene | -0.63333 | -76.15 | 213 | 502 | TF | 0.010131 | -0.10322 |
| TIP_03 | Mio-Pliocene | -0.63333 | -76.2333 | 103 | 334 | VA | 0.258572 | 0.000806 |
| TIP_04 | Mio-Pliocene | -0.61307 | -76.5335 | 147 | 1060 | SW | 0.145137 | -0.01686 |
| TIP_05 | Mio-Pliocene | -0.63243 | -76.15 | 222 | 663 | TF | 0.131158 | -0.19107 |
| TSUIRIM | Paleocene | -2.62639 | -77.8042 | 52 | 241 | TF | 0.014216 | 0.177391 |
| TUKUP | Pleistocene | -2.80278 | -77.4983 | 99 | 307 | TF | 0.069743 | -0.01174 |
| VILLANO | Pliocene | -1.47329 | -77.4095 | 131 | 319 | TF | 0.097306 | -0.04402 |
| WASURAK | Pleistocene | -2.57183 | -77.3612 | 139 | 206 | TF | 0.082427 | -0.11414 |
| YARINA | Pleistocene | -0.56306 | -75.23 | 158 | 632 | TF | 0.017815 | -0.03602 |
| YAS_JC | Pleistocene | -0.99167 | -75.45 | 96 | 430 | VA | 0.057799 | 0.136151 |
| YASUNI_JG | Mio-Pliocene | -0.55 | -76.0833 | 182 | 508 | VA | 0.177543 | -0.12686 |
| YUTURI | Pleistocene | -0.53333 | -76.05 | 203 | 476 | TF | 0.114562 | -0.18294 |
| ZANCUDO | Pleistocene | -0.617 | -75.3833 | 188 | 477 | TF | -0.13522 | -0.04232 |
| ZANCUDO_02 | Pleistocene | -0.579 | -75.4167 | 105 | 439 | TF | -0.34973 | 0.198749 |
| ZOJECHOE | Pleistocene | -0.32421 | -75.7005 | 179 | 433 | TF | -0.20988 | 0.013818 |
| ZOJECHOE_02 | Pleistocene | -0.33667 | -75.7 | 133 | 398 | TF | -0.2616 | 0.098336 |
|  |  |  |  |  |  |  |  |  |

Figure S1. A) Comparison of the observed values of phylogenetic turnover from a fully resolved phylogenetic tree from and the phylogenetic tree with polytomies at terminal nodes we used in this study.  The phylogeny of 931 species used in this comparison was derived from the phylogenetic tree published by Neves et al 2020. B) and C) Correlation between climatic and soils distances with overall phylogenetic beta diversity using the 931 species phylogeny generated from the phylogenetic tree published by Neves et al. 2020.


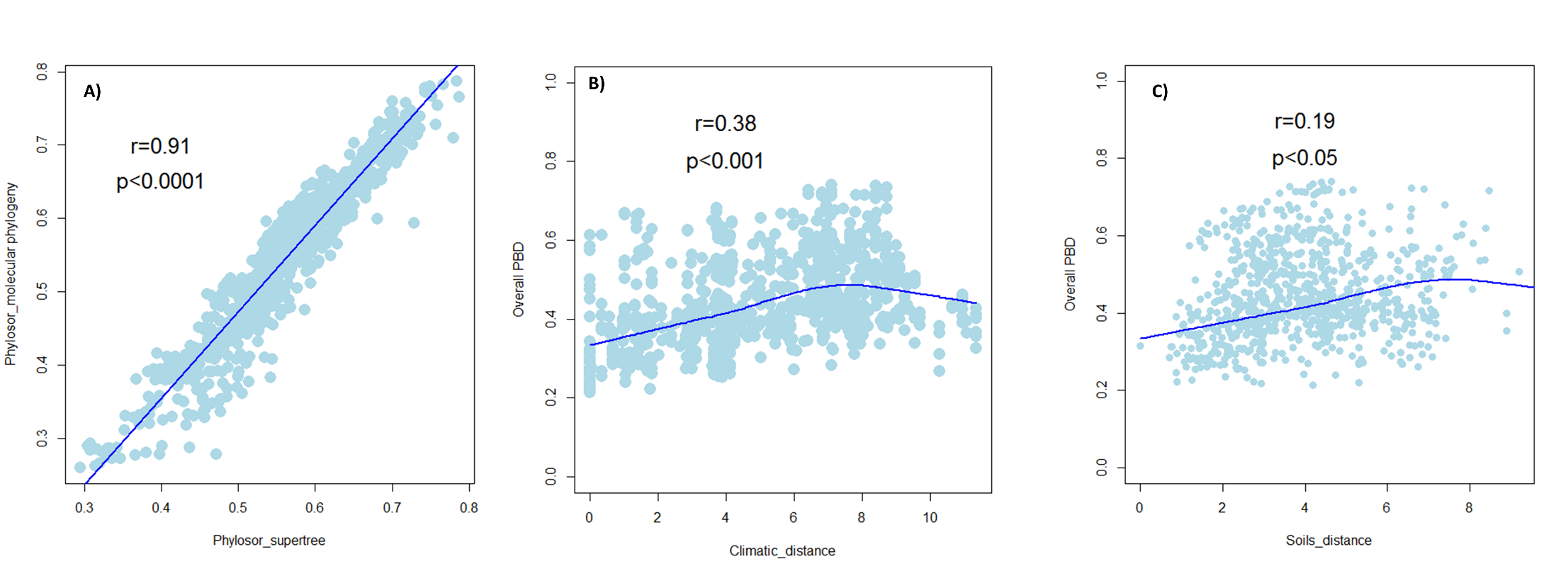


Figure S2. Distance Based Redundancy Analysis of 41 one hectare plots in the Ecuadorian Amazon; A) RDA analysis based on floristic composition and 8 soil variables with colored dots indicating the geological formation in which the plots are located; B) RDA analysis based on floristic composition and 19 climatic variables from the Worldclim database. Colored dots represent the floristic subregion the plots belongs to based on a hierarchical cluster analysis (Average method) using phylogenetic dissimilarity matrices.


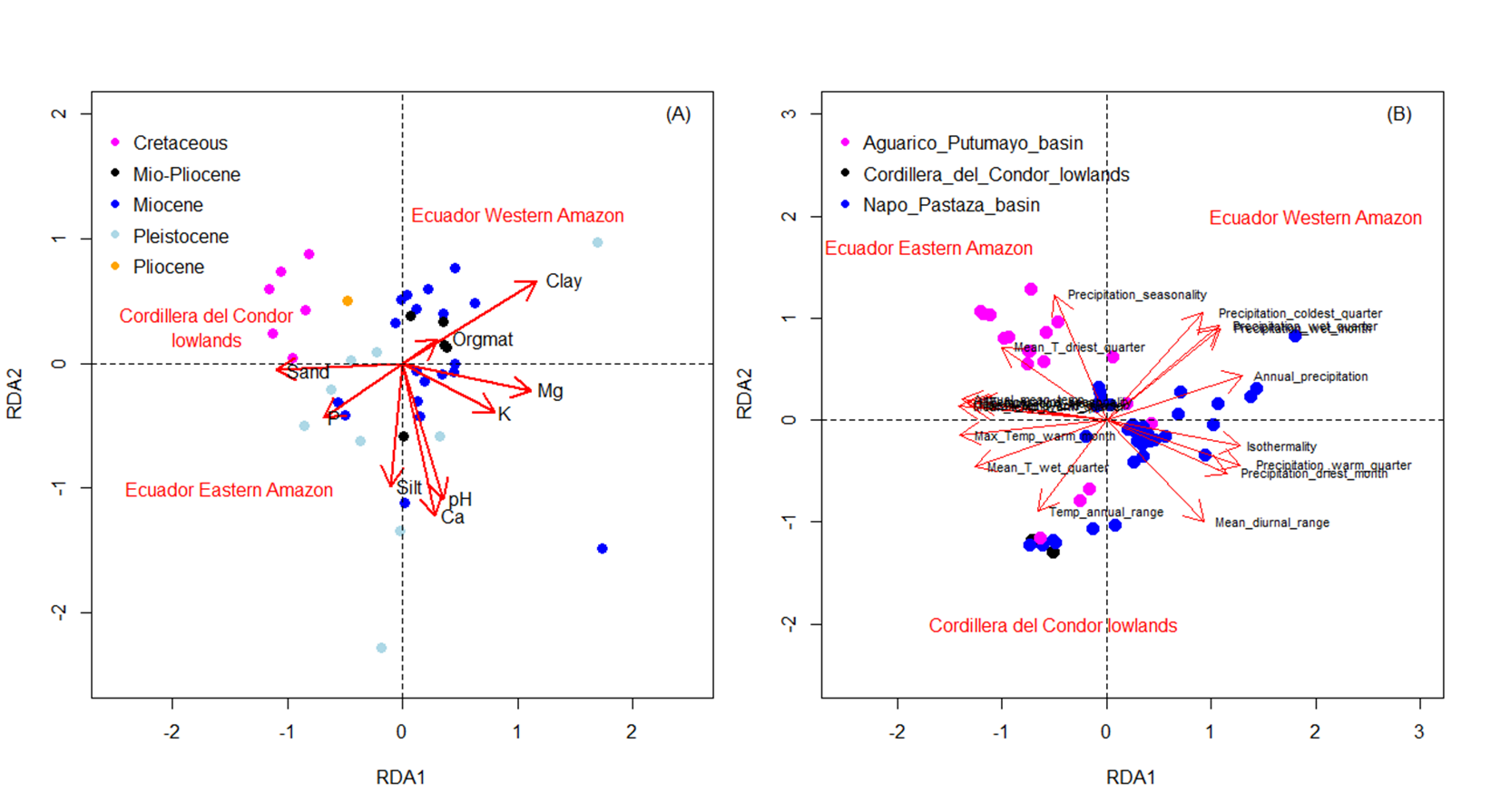


Figure S3. Climatic longitudinal gradient at regional scale in Ecuadorian Amazon. Climate is represented by the loadings from a Principal Component Analysis for 12 Worldclim climatic variables at 30 seconds spatial resolution; A) and B) correlations between longitude-latitude and annual precipitation (mm/yr) and average precipitation of the drier three months (dry season) respectively; C) spatial variation in precipitation for Ecuadorian Amazon; D) spatial variation in temperature for Ecuadorian Amazon. Interpolation of precipitation values was done using the kriging method on the basis of monthly mean precipitation values at 1 km2 resolution. Temperature values were interpolated using the kriging method on the basis of mean maximum and mean temperature values at 1 km2 resolution (Modified from *Forest Structure, Function and Dynamics in Western Amazonia* (41-43), by J.E. Guevara, USA: Wiley-Blackwell. Copyright 2017 by John Wiley & Sons Ltd. Reprinted with permission).


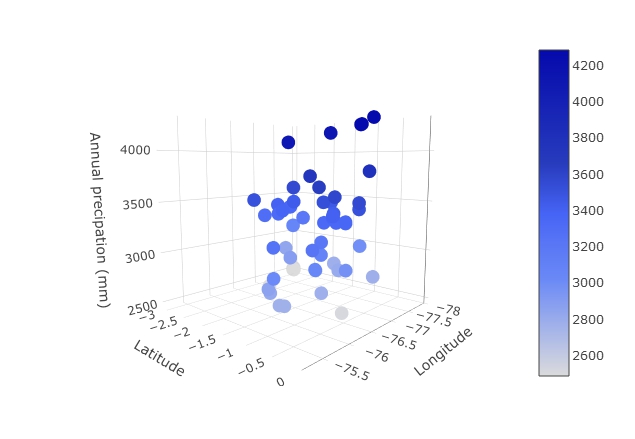

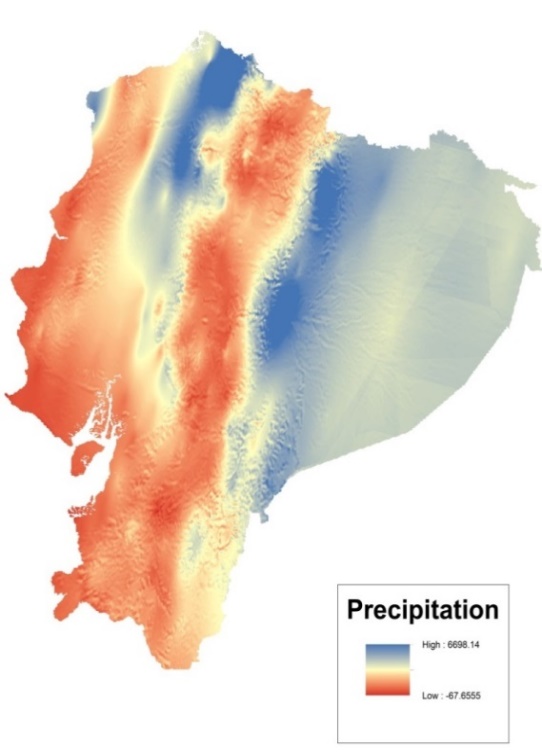

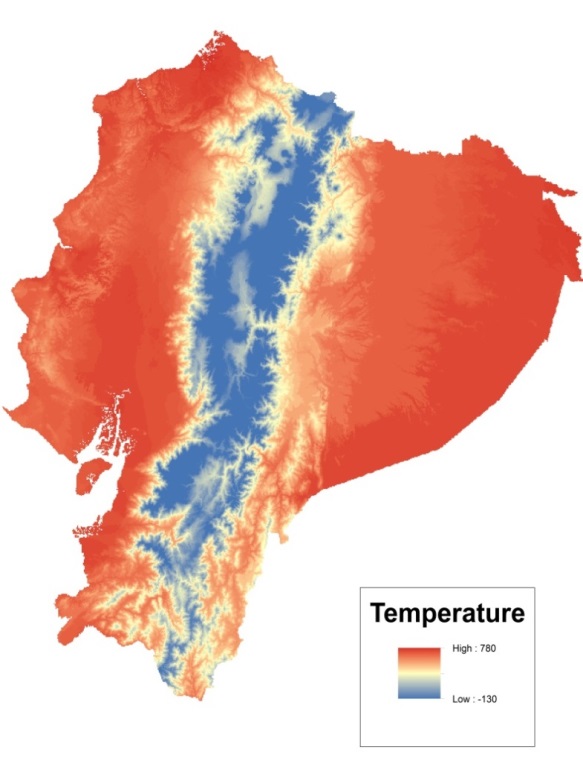


**C)**

**A)**

**D)**


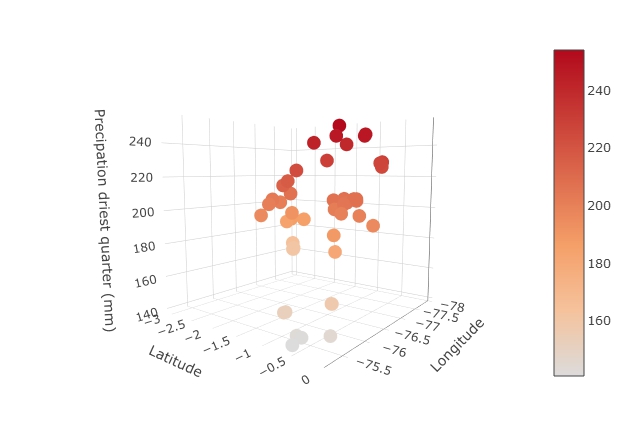


**B)**

Figure S4. Ultrametric phylogenetic tree for 1687 operational units present in a 40 one hectare plot network in Ecuadorian Amazon. Branch lengths represent millions of years.


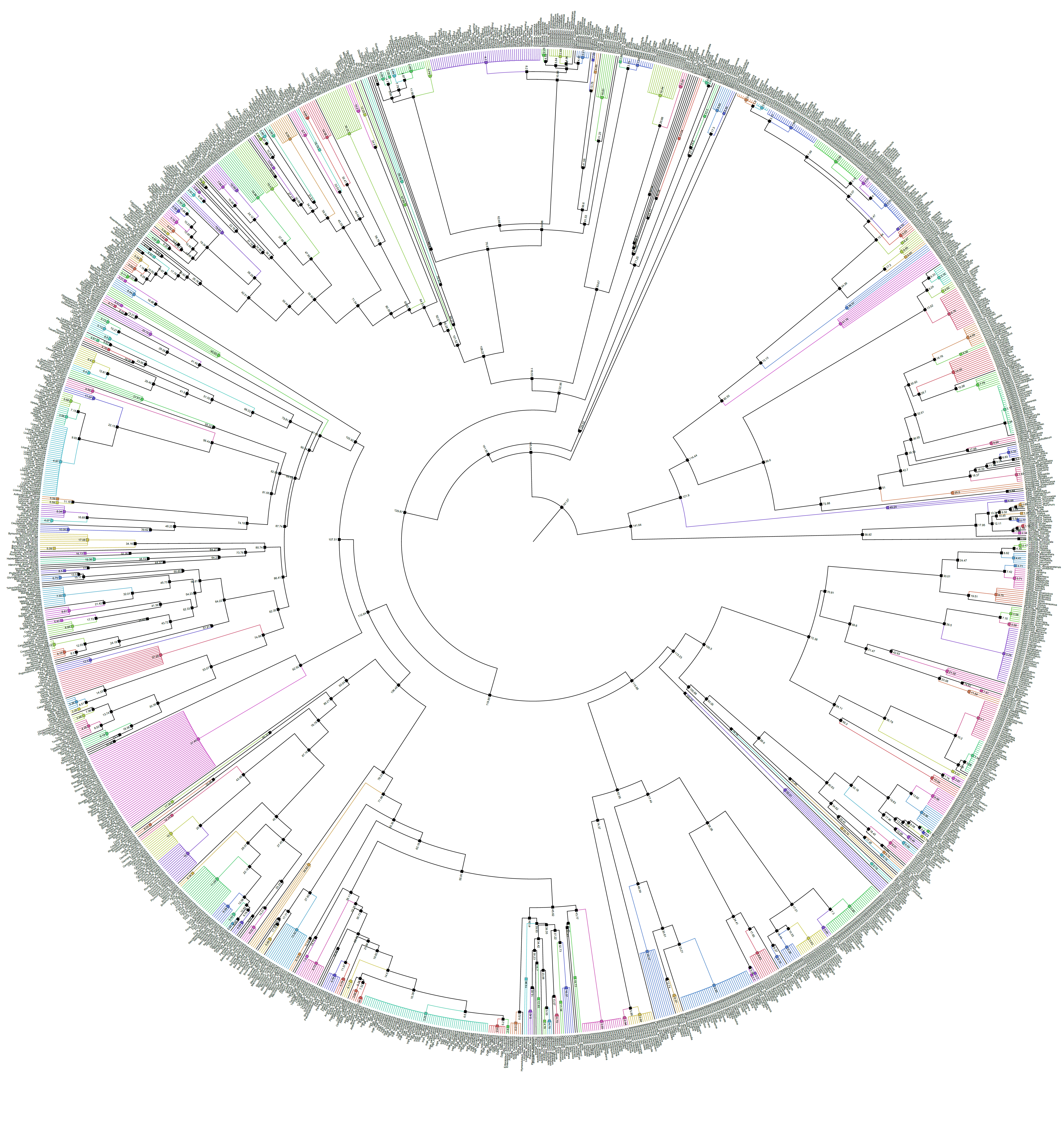


**Appendix S1**

(((((((((((((((Cupania_scrobiculata:2.16658,Cupania_burnham:2.16658,Cupania_cinerea:2.16658,Cupania_dentate:2.16658)Cupania:2.16658,Vouarana_anomala:4.33316):4.49033,(Matayba_guianensis:4.41174,Matayba_inelegans:4.41174,Matayba_peruviana:4.41174,Matayba_purgans:4.41174)Matayba:4.41174):15.6443,((Melicoccus_novogranatensis:3.70961,Melicoccus_oliviformis:3.70961)Melicoccus:3.70961,(Talisia_agria:3.70961,Talisia_cerasina:3.70961,Talisia_equis:3.70961,Talisia_gigantesca:3.70961,Talisia_megaphylla:3.70961,Talisia_microphylla:3.70961,Talisia_praealta:3.70961,Talisia_princeps:3.70961,Talisia_velvet:3.70961)Talisia:3.70961):17.0485):5.54488,((Allophylus_amazonicus:9.75337,Allophylus_divaricatus:9.75337,Allophylus_floribundus:9.75337,Allophylus_green:9.75337,Allophylus_pilosus:9.75337,Allophylus_punctatus:9.75337)Allophylus:9.75337,Paullinia_xestophylla:19.5067):10.5059):40.7953,((((Dacryodes_belemensis:3.57558,Dacryodes_chimantensis:3.57558,Dacryodes_gorda:3.57558,Dacryodes_nitens:3.57558,Dacryodes_peruviana:3.57558,Dacryodes_sclerophylla:3.57558)Dacryodes:3.57558,(Trattinnickia_boliviana:3.57558,Trattinnickia_glaziovii:3.57558,Trattinnickia_lawrancei:3.57558)Trattinnickia:3.57558):19.3437,(Protium_panamense:3.28816,Protium_paniculatum:3.28816,Protium_polybotryum:3.28816,Protium_rhoifolium:3.28816,Protium_rubrum:3.28816,Protium_sagotianum:3.28816,Protium_smooth:3.28816,Protium_subserratum:3.28816,Protium_tenuifolium:3.28816,Protium_trifoliolatum:3.28816,Protium_unifoliolatum:3.28816,Protium_aidanum:3.28816,Protium_altsonii:3.28816,Protium_amazonicum:3.28816,Protium_apiculatum:3.28816,Protium_aracouchini:3.28816,Protium_cuspi:3.28816,Protium_dacry:3.28816,Protium_divaricatum:3.28816,Protium_gallosum:3.28816,Protium_glabrescens:3.28816,Protium_guianense:3.28816,Protium_kaputna:3.28816,Protium_meridionale:3.28816,Protium_miniature:3.28816,Protium_nodulosum:3.28816,Protium_opacum:3.28816)Protium:23.2067):33.1049,((Spondias_mombin:42.6305,(Tapirira_gloomy:21.3153,Tapirira_guianensis:21.3153,Tapirira_immense:21.3153,Tapirira_obtusa:21.3153)Tapirira:21.3153):8.84137,((Astronium_graveolens:14.8175,(Thyrsodium_dik:7.40875,Thyrsodium_herrerense:7.40875)Thyrsodium:7.40875):8.26584,(Anacardium_blood:11.5417,Anacardium_excelsum:11.5417,Anacardium_giganteum:11.5417)Anacardium:11.5417):28.3886):8.12785):11.2082):1.5727,((((Trichilia_pleeana:5.09778,Trichilia_poeppigii:5.09778,Trichilia_quadrijuga:5.09778,Trichilia_rubra:5.09778,Trichilia_septentrionalis:5.09778,Trichilia_solitudinis:5.09778,Trichilia_walter:5.09778,Trichilia_adolfi:5.09778,Trichilia_cipo:5.09778,Trichilia_elegans:5.09778,Trichilia_elsae:5.09778,Trichilia_euneura:5.09778,Trichilia_inaequilatera:5.09778,Trichilia_laxipaniculata:5.09778,Trichilia_maynasiana:5.09778,Trichilia_micrantha:5.09778,Trichilia_obovata:5.09778,Trichilia_pallida:5.09778,Trichilia_pittieri:5.09778)Trichilia:5.09778,(((Guarea_carinata:1.53905,Guarea_cinnamomea:1.53905,Guarea_ecuadoriensis:1.53905,Guarea_fistulosa:1.53905,Guarea_glabra:1.53905,Guarea_gomma:1.53905,Guarea_grandifolia:1.53905,Guarea_guentheri:1.53905,Guarea_guidonia:1.53905,Guarea_kunthiana:1.53905,Guarea_macro:1.53905,Guarea_macrophylla:1.53905,Guarea_percy:1.53905,Guarea_pterorhachis:1.53905,Guarea_pubescens:1.53905,Guarea_purusana:1.53905,Guarea_silvatica:1.53905)Guarea:1.53905,Ruagea_insignis:3.0781):1.5176,Cabralea_canjerana:4.59569):5.59986):25.5921,(Cedrela_fissilis:5.21292,Cedrela_odorata:5.21292)Cedrela:30.5748):18.3237,(((Simaba_guianensis:3.86823,Simaba_orinocensis:3.86823,Simaba_paraensis:3.86823,Simaba_polyphylla:3.86823)Simaba:3.86823,Simarouba_amara:7.73646):41.5672,(Zanthoxylum_acuminatum:10.5385,Zanthoxylum_climb:10.5385,Zanthoxylum_formiciferum:10.5385,Zanthoxylum_riedelianum:10.5385,Zanthoxylum_sprucei:10.5385)Zanthoxylum:38.7652):4.80772):18.2693):33.1216,((((((((Sterculia_apeibophylla:6.96076,Sterculia_apetala:6.96076,Sterculia_colombiana:6.96076,Sterculia_frondosa:6.96076,Sterculia_guapayensis:6.96076,Sterculia_killipiana:6.96076,Sterculia_pruriens:6.96076,Sterculia_rebeccae:6.96076,Sterculia_tessmannii:6.96076)Sterculia:6.96076,(Matisia_idroboi:6.96076,Matisia_lasiocalyx:6.96076,Matisia_lomensis:6.96076,Matisia_longiflora:6.96076,Matisia_malacocalyx:6.96076,Matisia_obliquifolia:6.96076,Matisia_ochrocalyx:6.96076,Matisia_arteagensis:6.96076,Matisia_bracteolosa:6.96076,Matisia_cordata:6.96076)Matisia:6.96076):5.91003,(((((Eriotheca_globosa:1.12821,Eriotheca_macrophylla:1.12821)Eriotheca:3.2763,(Pachira_aquatica:2.20226,Pachira_insignis:2.20226)Pachira:2.20226):4.17074,(Ceiba_pentandra:1.78607,Ceiba_samauma:1.78607)Ceiba:6.78918):4.63164,Ochroma_pyramidale:13.2069):4.25301,((Quararibea_amazonica:5.4912,Quararibea_spatulata:5.4912,Quararibea_wittii:5.4912)Quararibea:5.4912,Pentaplaris_huaoranica:10.9824):6.47751):2.37165):13.3469,(Luehea_cymulosa:5.59467,Luehea_deciduous:5.59467,Luehea_white:5.59467)Luehea:27.5837):7.45128,(((Theobroma_cacao:9.21123,Theobroma_colonial:9.21123,Theobroma_glaucum:9.21123,Theobroma_speciosum:9.21123,Theobroma_subincanum:9.21123)Theobroma:9.21123,(Guazuma_ulmifolia:10.8922,Herrania_nitida:10.8922):7.5303):16.2019,(((Apeiba_aspera:8.74058,Apeiba_tibourbou:8.74058)Apeiba:8.74058,Lueheopsis_rosea:17.48116):11.9697,Heliocarpus_americanus:29.4509):5.1735):6.00533):28.9699,(Bixa_platycarpa:25.7411,Bixa_urucurana:25.7411)Bixa:43.8586):23.4526,(Capparis_detonsa:79.178,Jacaratia_digitata:79.17795):13.8742):7.62882,Huertea_glandulosa:100.681):4.82124):7.72869,((Picramnia_faboideae:50.008,Picramnia_juniniana:50.008,Picramnia_latifolia:50.008,Picramnia_spruceana:50.008)Picramnia:50.008,Turpinia_occidentalis:100.016):13.2149):2.65043,((((((Eugenia_egensis:3.6489,Eugenia_feijoi:3.6489,Eugenia_florida:3.6489,Eugenia_galalonensis:3.6489,Eugenia_heterochroma:3.6489,Eugenia_kaputna:3.6489,Eugenia_lambertiana:3.6489,Eugenia_lime:3.6489,Eugenia_macrocalyx:3.6489,Eugenia_margin:3.6489,Eugenia_marowynensis:3.6489,Eugenia_multirimosa:3.6489,Eugenia_muricata:3.6489,Eugenia_patens:3.6489,Eugenia_patrisii:3.6489,Eugenia_pinprick:3.6489,Eugenia_schunkei:3.6489,Eugenia_soft:3.6489,Eugenia_sovran:3.6489,Eugenia_stipitata:3.6489,Eugenia_tetrasticha:3.6489,Eugenia_yasuniana:3.6489,Eugenia_anastomosans:3.6489,Eugenia_black:3.6489,Eugenia_blacksheep:3.6489,Eugenia_chicaretic:3.6489,Eugenia_cuspidifolia:3.6489,Eugenia_dittocrepis:3.6489,Eugenia_doublering:3.6489)Eugenia:3.6489,(Plinia_myro:3.6489,Plinia_tan:3.6489)Plinia:3.6489):9.7128,(((Calyptranthes_rabbit:3.62791,Calyptranthes_ruiziana:3.62791,Calyptranthes_salacia:3.62791,Calyptranthes_tessmannii:3.62791,Calyptranthes_bipennis:3.62791,Calyptranthes_branch:3.62791,Calyptranthes_brosimoid:3.62791,Calyptranthes_cortezanegra:3.62791,Calyptranthes_doradita:3.62791,Calyptranthes_macrophylla:3.62791,Calyptranthes_maxima:3.62791,Calyptranthes_mistic:3.62791,Calyptranthes_nearbranch:3.62791,Calyptranthes_paniculata:3.62791)Calyptranthes:6.89783,(Myrcia_aliena:5.26287,Myrcia_chiquita:5.26287,Myrcia_guianensis:5.26287,Myrcia_portuguese:5.26287,Myrcia_ridgely:5.26287,Myrcia_splendens:5.26287)Myrcia:5.26287):1.61488,(Psidium_acutangulum:8.37098,(Myrciaria_amazonica:4.18549,Myrciaria_dubia:4.18549,Myrciaria_dubius:4.18549,Myrciaria_floribunda:4.18549)Myrciaria:4.18549):3.76964):4.86997):41.2494,((Erisma_uncinatum:17.6521,(Vochysia_aff_grandis:8.82605,Vochysia_biloba:8.82605,Vochysia_braceliniae:8.82605,Vochysia_ferruginea:8.82605,Vochysia_floribunda:8.82605,Vochysia_grandis:8.82605,Vochysia_lomatophylla:8.82605,Vochysia_splendens:8.82605,Vochysia_vismiifolia:8.82605)Vochysia:8.82605):6.76229,((Qualea_acuminata:2.69639,Qualea_paraensis:2.69639)Qualea:2.69639,(Ruizterania_cassiquiarensis:2.69639,Ruizterania_trichanthera:2.69639)Ruizterania:2.69639):19.0216):33.8456):19.1808,(((Miconia_lamprophylla:4.9456,Miconia_longifolia:4.9456,Miconia_minutiflora:4.9456,Miconia_multispicata:4.9456,Miconia_napoana:4.9456,Miconia_oval:4.9456,Miconia_pilgeriana:4.9456,Miconia_prasina:4.9456,Miconia_pterocaulon:4.9456,Miconia_punctata:4.9456,Miconia_rust:4.9456,Miconia_saramago:4.9456,Miconia_splendens:4.9456,Miconia_subspicata:4.9456,Miconia_tipica5ha:4.9456,Miconia_trinervia:4.9456,Miconia_turku:4.9456,Miconia_abbreviata:4.9456,Miconia_ampla:4.9456,Miconia_bubalina:4.9456,Miconia_cazaletii:4.9456,Miconia_cernua:4.9456,Miconia_white:4.9456,Miconia_decurrens:4.9456,Miconia_elata:4.9456,Miconia_estrobico:4.9456,Miconia_flask:4.9456,Miconia_france:4.9456,Miconia_glaucescens:4.9456,Miconia_grandifolia:4.9456,Miconia_insularis:4.9456,Miconia_klugii:4.9456)Miconia:25.66019,(Bellucia_pentamera:6.3689,Bellucia_subandina:6.3689)Bellucia:24.2369):17.9363,(Mouriri_icarus:24.2711,Mouriri_laxiflora:24.2711,Mouriri_myrtilloides:24.2711,Mouriri_nigra:24.2711,Mouriri_oligantha:24.2711,Mouriri_vernicosa:24.2711,Mouriri_acutiflora:24.2711)Mouriri:24.2711):28.8986):5.50859,(Lafoensia_acuminata:74.3728,((Buchenavia_congesta:2.94339,Buchenavia_grandis:2.94339,Buchenavia_macrophylla:2.94339,Buchenavia_oxycarpa:2.94339,Buchenavia_parvifolia:2.94339,Buchenavia_reticulata:2.94339,Buchenavia_seri:2.94339,Buchenavia_sericocarpa:2.94339,Buchenavia_suaveolens:2.94339,Buchenavia_viridiflora:2.94339,Buchenavia_amazonia:2.94339)Buchenavia:2.94339,(Terminalia_amazonia:2.94339,Terminalia_oblonga:2.94339)Terminalia:2.94339):68.486):8.57651):32.9321):3.96577,(((((((((Swartzia_rojitaseri:3.02902,Swartzia_arborescens:3.02902,Swartzia_benthamiana:3.02902,Swartzia_bombycina:3.02902,Swartzia_cardiosperma:3.02902,Swartzia_cuspi:3.02902,Swartzia_cuspidata:3.02902,Swartzia_falsatangarana:3.02902,Swartzia_feeble:3.02902,Swartzia_laevicarpa:3.02902,Swartzia_macrosema:3.02902,Swartzia_multijuga:3.02902,Swartzia_myrtifolia:3.02902,Swartzia_polyphylla:3.02902,Swartzia_racemosa:3.02902,Swartzia_reticulata:3.02902)Swartzia:40.7434,((Dussia_gorda:19.7171,Dussia_tessmannii:19.7171)Dussia:19.7171,(Myroxylon_balsamum:37.9262,Dipteryx_micrantha:37.9262):1.508):4.33817):1.84923,((((((Ormosia_paraensis:16.3668,Ormosia_amazonica:16.3668,Ormosia_elata:16.3668,Ormosia_kaputna:16.3668)Ormosia:16.3668,(Diplotropis_martiusii:11.3383,Diplotropis_purpurea:11.3383)Diplotropis:21.3952):4.48894,(Vatairea_erythrocarpa:6.78702,Vatairea_fusca:6.78702,Vatairea_guianensis:6.78702)Vatairea:30.43542):2.13208,((((Machaerium_aristulatum:4.77555,Machaerium_floribundum:4.77555,Machaerium_leiophyllum:4.77555)Machaerium:4.77555,(Dalbergia_frutescens:4.77555,Dalbergia_monetaria:4.77555)Dalbergia:4.77555):13.507,Platymiscium_stipulare:23.0581):11.3644,(((Erythrina_amazonica:12.8521,Erythrina_poeppigiana:12.8521)Erythrina:12.8521,Clitoria_arborea:25.7042):4.52388,(Lonchocarpus_seorsus:16.8476,(Pterocarpus_amazonum:8.4238,Pterocarpus_brown:8.4238,Pterocarpus_rohrii:8.4238)Pterocarpus:8.4238):13.3804):4.19444):4.93207):0.574325,Lecointea_peruviana:39.9288):1.91436,(Andira_inermis:4.01047,Andira_macrocarpa:4.01047,Andira_macrothyrsa:4.01047,Andira_multistipula:4.01047,Andira_surinamensis:4.01047)Andira:37.83277):3.7784):10.2844,((((((((((Enterolobium_schomburgkii:5.19872,(Zygia_coccinea:2.59936,Zygia_heteroneura:2.59936,Zygia_inaequalis:2.59936,Zygia_juruana:2.59936,Zygia_lathetica:2.59936,Zygia_unexpected:2.59936)Zygia:2.59936):1.3048,(Inga_yarina:3.25176,Inga_yasuniana:3.25176,Inga_heterophylla:3.25176,Inga_ilta:3.25176,Inga_leiocalycina:3.25176,Inga_lenticelosa:3.25176,Inga_marginata:3.25176,Inga_multijuga:3.25176,Inga_multinervis:3.25176,Inga_nobilis:3.25176,Inga_oerstediana:3.25176,Inga_paraensis:3.25176,Inga_pezizifera:3.25176,Inga_poeppigiana:3.25176,Inga_pruriens:3.25176,Inga_psittacorum:3.25176,Inga_punctata:3.25176,Inga_3oscura:3.25176,Inga_ruiziana:3.25176,Inga_acreana:3.25176,Inga_rusbyi:3.25176,Inga_acuminata:3.25176,Inga_alata:3.25176,Inga_sapindoides:3.25176,Inga_sarayacuensis:3.25176,Inga_alavelu:3.25176,Inga_alba:3.25176,Inga_sertulifera:3.25176,Inga_auristellae:3.25176,Inga_spectabilis:3.25176,Inga_bourgoni:3.25176,Inga_splendens:3.25176,Inga_brachyrhachis:3.25176,Inga_stipulacea:3.25176,Inga_capitata:3.25176,Inga_cayennensis:3.25176,Inga_suaveolens:3.25176,Inga_chartacea:3.25176,Inga_ciliata:3.25176,Inga_tenuistipula:3.25176,Inga_tessmannii:3.25176,Inga_cinnamomea:3.25176,Inga_thibaudiana:3.25176,Inga_cordatoalata:3.25176,Inga_umbellifera:3.25176,Inga_coruscans:3.25176,Inga_umbratica:3.25176,Inga_cylindrica:3.25176,Inga_velutina:3.25176,Inga_delgadaoerst:3.25176,Inga_venusta:3.25176,Inga_vera:3.25176,Inga_edulis:3.25176,Inga_vismiifolia:3.25176,Inga_glomeriflora:3.25176,Inga_yacoana:3.25176,Inga_gracilifolia:3.25176,Inga_gracilior:3.25176)Inga:3.25176):3.88991,(((Abarema_adenophora:1.91481,Abarema_jupunba:1.91481)Abarema:5.4041,Albizia_niopoides:7.31892):0.966205,Cedrelinga_cateniformis:8.28512):2.10831):4.1022,(Calliandra_guildingii:6.11176,Calliandra_large:6.11176,Calliandra_trinervia:6.11176)Calliandra:8.38387):5.32253,Piptadenia_pteroclada:19.8182):2.02193,((Acacia_immense:5.78636,Acacia_multipinnata:5.78636)Acacia:5.78636,(Parkia_balslevii:5.78636,Parkia_igneiflora:5.78636,Parkia_multijuga:5.78636,Parkia_nitida:5.78636,Parkia_pendula:5.78636,Parkia_velutina:5.78636)Parkia:5.78636):10.2674):0.976015,(Pseudopiptadenia_suaveolens:14.9408,Stryphnodendron_porcatum:14.9408):7.87533):7.89955,Schizolobium_parahyba:30.7156):1.5362,(Tachigali_chrysophylla:6.75327,Tachigali_formicarum:6.75327,Tachigali_inconspicua:6.75327,Tachigali_paniculata:6.75327,Tachigali_paraensis:6.75327,Tachigali_ptychophysca:6.75327,Tachigali_setifera:6.75327)Tachigali:25.4986):2.86052,(Senna_trolliiflora:14.79294,Cassia_cowanii:14.7929):20.3194):20.7937):6.25739,(Dialium_guianense:13.5869,Apuleia_ferruginea:13.58694):48.5765):3.18282,((Macrolobium_acaciifolium:13.815,Macrolobium_angustifolium:13.815,Macrolobium_archeri:13.815,Macrolobium_bifolium:13.815,Macrolobium_colombianum:13.815,Macrolobium_gracile:13.815,Macrolobium_ischnocalyx:13.815,Macrolobium_limbatum:13.815,Macrolobium_microcalyx:13.815,Macrolobium_multijugum:13.815,Macrolobium_novel:13.815,Macrolobium_stenocladum:13.815,Macrolobium_suaveolens:13.815)Macrolobium:13.815,(Hymenaea_oblongifolia:17.7683,(((Brownea_grandiceps:5.8901,Brownea_lore:5.8901,Brownea_macrophylla:5.8901)Brownea:5.8901,Crudia_glaberrima:11.7802):1.86736,Browneopsis_ucayalina:13.6476):4.12069):9.86182):37.7162):6.30873,(Bauhinia_arborea:35.8275,Bauhinia_brachycalyx:35.8275,Bauhinia_tarapotensis:35.8275)Bauhinia:35.8275):34.8226,(((((((((Sorocea_guilleminiana:6.01731,Sorocea_steinbachii:6.01731)Sorocea:7.99461,Trophis_caucana:14.0119):11.237,(Batocarpus_orinocensis:9.02039,Clarisia_racemosa:9.02039):16.2285):12.1802,((((((Perebea_guianensis:4.88835,Perebea_mollis:4.88835,Perebea_tessmannii:4.88835)Perebea:4.88835,(Pseudolmedia_rigida:4.95101,(Maquira_calophylla:2.47551,Maquira_guianensis:2.47551)Maquira:2.47551):4.82569):1.07067,(Helicostylis_elegans:5.42369,Helicostylis_scabra:5.42369,Helicostylis_turbinata:5.42369)Helicostylis:5.42369):1.7338,(Naucleopsis_concinna:6.29058,Naucleopsis_glabra:6.29058,Naucleopsis_herrerensis:6.29058,Naucleopsis_humilis:6.29058,Naucleopsis_imitans:6.29058,Naucleopsis_oblongifolia:6.29058,Naucleopsis_ternstroemiiflora:6.29058)Naucleopsis:6.29058):9.5956,(Ficus_brevibracteata:11.0884,Ficus_cuatrecasasiana:11.0884,Ficus_guianensis:11.0884,Ficus_juno:11.0884,Ficus_mathewsii:11.0884,Ficus_maxima:11.0884,Ficus_piresiana:11.0884,Ficus_schippii:11.0884,Ficus_trigona:11.0884,Ficus_trigonata:11.0884,Ficus_uiant:11.0884,Ficus_ursina:11.0884)Ficus:11.0884):5.89717,(Brosimum_acutifolium:6.34338,Brosimum_potabile:6.34338)Brosimum:21.7306):9.35516):4.56628,Maclura_tinctoria:41.9954):25.1642,(((Pourouma_minor:9.99766,Pourouma_mollis:9.99766,Pourouma_myrmecophila:9.99766,Pourouma_napoensis:9.99766,Pourouma_petiolulata:9.99766,Pourouma_tomentosa:9.99766,Pourouma_acuminata:9.99766,Pourouma_bicolor:9.99766,Pourouma_cecropiifolia:9.99766,Pourouma_cucura:9.99766,Pourouma_defiant:9.99766,Pourouma_guianensis:9.99766,Pourouma_melinonii:9.99766)Pourouma:9.99766,(Cecropia_angustifolia:9.99766,Cecropia_distachya:9.99766,Cecropia_engleriana:9.99766,Cecropia_ficifolia:9.99766,Cecropia_herthae:9.99766,Cecropia_hispid:9.99766,Cecropia_latiloba:9.99766,Cecropia_membranacea:9.99766,Cecropia_peru:9.99766,Cecropia_putumayonis:9.99766,Cecropia_sciadophylla:9.99766)Cecropia:9.99766):23.5654,Urera_caracasana:43.5608):23.5989):9.62213,(Celtis_schippii:33.9511,(Ampelocera_edentula:6.04683,Ampelocera_longissima:6.04683)Ampelocera:27.9043):42.8306):8.42784,(Rhamnidium_elaeocarpum:60.3181,Colubrina_arborescens:60.318):24.8915):8.33157,Prunus_debilis:93.5411):12.9365):6.16433,(((Sloanea_eichleri:37.4933,Sloanea_erismoides:37.4933,Sloanea_falsebark:37.4933,Sloanea_family:37.4933,Sloanea_floribunda:37.4933,Sloanea_fragante:37.4933,Sloanea_froesii:37.4933,Sloanea_grandiflora:37.4933,Sloanea_granulosa:37.4933,Sloanea_guianensis:37.4933,Sloanea_hirtella:37.4933,Sloanea_lasiocoma:37.4933,Sloanea_laurifolia:37.4933,Sloanea_longif:37.4933,Sloanea_macrophylla:37.4933,Sloanea_meianthera:37.4933,Sloanea_monosperma:37.4933,Sloanea_multiflora:37.4933,Sloanea_obtusifolia:37.4933,Sloanea_opposite:37.4933,Sloanea_oppositifolia:37.4933,Sloanea_peculiar:37.4933,Sloanea_pinguino:37.4933,Sloanea_pubescens:37.4933,Sloanea_robusta:37.4933,Sloanea_sinemariensis:37.4933,Sloanea_bark:37.4933,Sloanea_brachytepala:37.4933,Sloanea_brevipes:37.4933,Sloanea_catostemoide:37.4933,Sloanea_cordia:37.4933,Sloanea_durissima:37.4933,Sloanea_spathulata:37.4933,Sloanea_stipi:37.4933,Sloanea_synandra:37.4933,Sloanea_tuerckheimii:37.4933,Sloanea_ugly:37.4933)Sloanea:45.0246,Connarus_dubia:82.5179):24.9925,((((((((((((Symphonia_globulifera:13.2014,Moronobea_coccinea:13.2014):5.17815,(Garcinia_brasiliensis:9.18977,Garcinia_condor:9.18977,Garcinia_intermedia:9.18977,Garcinia_macrophylla:9.18977,Garcinia_madruno:9.18977)Garcinia:9.18977):11.9792,((Dystovomita_brasiliensis:8.52662,(Tovomita_calophyllophylla:4.26331,Tovomita_choisyana:4.26331,Tovomita_grata:4.26331,Tovomita_macrophylla:4.26331,Tovomita_mitopsis:4.26331,Tovomita_umbellata:4.26331,Tovomita_weddelliana:4.26331)Tovomita:4.26331):5.2087,((Chrysochlamys_intersecpromin:3.98015,Chrysochlamys_membranacea:3.98015)Chrysochlamys:3.98015,Clusia_amazonica:7.9603):5.77502):16.6234):22.7071,((Caraipa_densifolia:6.51354,(Marila_alternifolia:3.25677,Marila_myrsinac:3.25677,Marila_pluricostata:3.25677,Marila_tomentosa:3.25677)Marila:3.25677):7.81586,Calophyllum_brasiliense:14.3294):38.7365):21.4979,(Vismia_baccifera:37.2819,Vismia_lauriformis:37.2819,Vismia_lemonade:37.2819,Vismia_macrophylla:37.2819,Vismia_myrsinac:37.2819,Vismia_palate:37.2819,Vismia_punctate:37.2819,Vismia_sprucei:37.2819,Vismia_weedy:37.2819)Vismia:37.2819):7.78703,(((Pera_benensis:12.4991,Pera_bicolor:12.4991)Pera:45.309,Pogonophora_schomburgkiana:57.8082):6.40663,(((((Alchorneopsis_floribunda:24.1899,((Aparisthmium_cordatum:8.30028,(Alchornea_discolor:4.15014,Alchornea_glandulosa:4.15014,Alchornea_latifolia:4.15014,Alchornea_triplinervia:4.15014,Alchornea_webster:4.15014)Alchornea:4.15014):3.72841,(Conceveiba_guianensis:1.1512,Conceveiba_rhytidocarpa:1.1512,Conceveiba_split:1.1512,Conceveiba_terminalis:1.1512)Conceveiba:10.8775):12.1612):19.5277,Caryodendron_orinocense:43.71764):8.61409,(((Croton_cuneatus:8.8596,Croton_lechleri:8.8596,Croton_matourensis:8.8596,Croton_tessmannii:8.8596,Croton_yellow:8.8596)Croton:8.8596,(Sagotia_brachysepala:5.42111,Sagotia_racemosa:5.42111)Sagotia:12.2981):23.6732,Pausandra_trianae:41.3924):10.9394):1.89802,((((Sapium_marmieri:8.60554,Sapium_ciliatum:8.60554,Sapium_garcinia:8.60554,Sapium_glandulosum:8.60554)Sapium:12.8156,Hura_crepitans:21.4211):10.589,(Mabea_acutissima:7.5453,Mabea_angularis:7.5453,Mabea_klugii:7.5453,Mabea_macbridei:7.5453,Mabea_nitida:7.5453,Mabea_piriri:7.5453,Mabea_speciosa:7.5453,Mabea_subsessilis:7.5453)Mabea:24.4648):13.7202,Nealchornea_yapurensis:45.7303):8.49944):2.38115,(Tetrorchidium_macrophyllum:50.8885,(Hevea_guianensis:15.1685,((Micrandra_rossiana:6.7512,Micrandra_spruceana:6.7512)Micrandra:6.7512,Glycydendron_amazonicum:13.5024):1.6661):35.72):5.72242):7.6039):18.136):4.05426,(((((Phyllanthus_attenuatus:16.99812,Margaritaria_nobilis:16.9981):27.3698,Amanoa_guianensis:44.3679):19.8323,(Richeria_grandis:38.7167,(Hieronyma_alchorneoides:19.3584,Hieronyma_kaputna:19.3584,Hieronyma_oblonga:19.3584)Hieronyma:19.3584):25.4835):9.58283,(Hebepetalum_humiriifolium:32.2601,Roucheria_schomburgkii:32.2602):41.52293):6.95687,((Bunchosia_argentea:5.08962,Bunchosia_hookeriana:5.08962)Bunchosia:29.066,(Byrsonima_arthropoda:17.0778,Byrsonima_densa:17.0778,Byrsonima_japurensis:17.0778,Byrsonima_kampa:17.0778,Byrsonima_otra:17.0778,Byrsonima_putumayensis:17.0778)Byrsonima:17.0778):46.5843):5.66514):1.33251,(((((Ouratea_pendula:39.623,Cespedesia_spathulata:39.6229):8.60249,((Lacunaria_jenmanii:4.06569,Lacunaria_crenata:4.06569,Lacunaria_green:4.06569)Lacunaria:12.6199,(Quiina_amazonica:8.34281,Quiina_aulestia:8.34281,Quiina_florida:8.34281,Quiina_macrophylla:8.34281)Quiina:8.34281):31.5398):25.9582,((Caryocar_glabrum:5.58835,Caryocar_villosum:5.58835)Caryocar:5.58835,Anthodiscus_klugii:11.1767):63.0069):7.36849,((((Licania_latifolia:4.96548,Licania_longipedicellata:4.96548,Licania_longistyla:4.96548,Licania_macrocarpa:4.96548,Licania_micrantha:4.96548,Licania_octandra:4.96548,Licania_pallida:4.96548,Licania_reticulata:4.96548,Licania_silvae:4.96548,Licania_sothersiae:4.96548,Licania_triandra:4.96548,Licania_unguiculata:4.96548,Licania_urceolaris:4.96548,Licania_velutina:4.96548,Licania_arborea:4.96548,Licania_bitterlime:4.96548,Licania_blackii:4.96548,Licania_brittoniana:4.96548,Licania_canescens:4.96548,Licania_caudata:4.96548,Licania_cuyabenensis:4.96548,Licania_durifolia:4.96548,Licania_egleri:4.96548,Licania_elliptica:4.96548,Licania_granvillei:4.96548,Licania_guianensis:4.96548,Licania_harlingii:4.96548,Licania_heteromorpha:4.96548,Licania_hypoleuca:4.96548,Licania_kaputna:4.96548,Licania_krukovii:4.96548,Licania_kunthiana:4.96548,Licania_lata:4.96548)Licania:4.96548,((Hirtella_excelsa:3.57646,Hirtella_macrophylla:3.57646,Hirtella_magnifolia:3.57646,Hirtella_pilosissima:3.57646,Hirtella_racemosa:3.57646,Hirtella_triandra:3.57646,Hirtella_aequatoriensis:3.57646,Hirtella_bicornis:3.57646,Hirtella_elongata:3.57646)Hirtella:3.57646,(Couepia_chrysocalyx:3.57646,Couepia_guianensis:3.57646,Couepia_kaputna:3.57646,Couepia_macrophylla:3.57646,Couepia_obovata:3.57646,Couepia_parillo:3.57646,Couepia_subcordata:3.57646)Couepia:3.57646):2.77804):12.2151,(Parinari_klugii:11.073,Parinari_neartin:11.073,Parinari_parilis:11.073)Parinari:11.073):34.2959,(Tapura_peruviana:9.88363,Tapura_amazonica:9.88363,Tapura_juruana:9.88363,Tapura_large:9.88363)Tapura:46.5583):25.1101):1.33365,(Sterigmapetalum_obovatum:55.2187,(Erythroxylum_citrifolium:27.6094,Erythroxylum_divaricatum:27.6094,Erythroxylum_macrophyllum:27.6094)Erythroxylum:27.6094):27.667):4.85182):0.913044,(((((((Laetia_corymbulosa:6.40437,Laetia_procera:6.40437,Laetia_suaveolens:6.40437)Laetia:6.40437,(Casearia_arborea:6.40437,Casearia_combaymensis:6.40437,Casearia_decandra:6.40437,Casearia_javitensis:6.40437,Casearia_sylvestris:6.40437,Casearia_tubiflora:6.40437,Casearia_uleana:6.40437,Casearia_ulmifolia:6.40437)Casearia:6.40437):16.6412,Lunania_parviflora:29.45):12.4463,Tetrathylacium_macrophyllum:41.8963):9.12145,((Homalium_racemosum:19.5555,(Xylosma_dubia:9.77774,Xylosma_intermedia:9.77774)Xylosma:9.77774):4.39434,((Hasseltia_floribunda:4.81323,Hasseltia_hasseltomen:4.81323)Hasseltia:4.81323,Pleuranthodendron_lindenii:9.62645):14.3234):27.068):15.2004,(Lacistema_aggregatum:8.49827,Lacistema_med:8.49827,Lacistema_nena:8.49827)Lacistema:57.7199):13.5909,(((Leonia_crassa:5.13412,Leonia_cymosa:5.13412,Leonia_glycycarpa:5.13412,Leonia_racemosa:5.13412)Leonia:5.13412,Gloeospermum_longifolium:10.26824):31.2224,(Rinorea_apiculata:20.7453,Rinorea_flavescens:20.7453,Rinorea_lindeniana:20.7453,Rinorea_viridifolia:20.7453)Rinorea:20.7453):38.3185):8.84151):1.59388,((Humiriastrum_diguense:9.5307,(Vantanea_parviflora:4.76535,Vantanea_peruviana:4.76535)Vantanea:4.76535):3.73509,(Sacoglottis_amazonica:6.63289,Sacoglottis_guianensis:6.63289)Sacoglottis:6.63289):76.9787):1.40688,(Drypetes_amazonica:45.8257,Drypetes_fanshawei:45.8257,Drypetes_heisteria:45.8257,Drypetes_variabilis:45.8257)Drypetes:45.8257):12.2705,(Maytenus_amazonica:8.44098,Maytenus_cuero:8.44098,Maytenus_guyanensis:8.44098,Maytenus_macrocarpa:8.44098,Maytenus_peking:8.44098)Maytenus:95.48088):3.5885):5.13155):7.2052):10.0181,(((((((((((((((((((((Duroia_eriopila:3.11039,Duroia_hirsuta:3.11039)Duroia:3.11039,Amaioua_corymbosa:6.22077):3.06951,((Alibertia_edulis:3.58925,Alibertia_isernii:3.58925,Alibertia_itayensis:3.58925,Alibertia_verrucosa:3.58925)Alibertia:3.58925,(Borojoa_claviflora:3.58925,Borojoa_fruits:3.58925,Borojoa_twin:3.58925)Borojoa:3.58925):2.11179):1.63137,Botryarrhena_pendula:10.92165):2.74534,(Kutchubaea_semisericea:6.8335,Kutchubaea_sericantha:6.8335)Kutchubaea:6.8335):4.12764,(Tocoyena_williamsii:8.83854,Randia_armata:8.83854):8.95609):2.82855,Genipa_americana:20.6232):5.13679,Ixora_killipii:25.76):16.7533,(((((Ferdinandusa_guainiae:4.62404,Ferdinandusa_uaupensis:4.62404,Ferdinandusa_elliptica:4.62404)Ferdinandusa:4.62404,Capirona_decorticans:9.24808):8.73812,(((Warszewiczia_cordata:6.43551,Warszewiczia_schwackei:6.43551)Warszewiczia:6.43551,(Calycophyllum_obovatum:5.45012,Calycophyllum_spruceanum:5.45012)Calycophyllum:7.42089):2.98033,((Alseis_dosel:6.32436,Alseis_lugonis:6.32436)Alseis:6.32436,((Simira_cordifolia:5.11586,Simira_harmful:5.11586,Simira_kampa:5.11586,Simira_wurdackii:5.11586)Simira:5.11586,(Pentagonia_amazonica:2.92027,Pentagonia_macrophylla:2.92027,Pentagonia_spathicalyx:2.92027)Pentagonia:7.31144):2.417):3.20263):2.13486):1.35064,Chimarrhis_gentryana:19.3368):19.9515,(Posoqueria_latifolia:19.6442,Posoqueria_panamensis:19.6442)Posoqueria:19.6442):3.22491):12.925,((((((Ladenbergia_acutifolia:2.80988,Ladenbergia_amazonensis:2.80988,Ladenbergia_muzonensis:2.80988)Ladenbergia:4.14107,(Remijia_chelomaphylla:3.47547,Remijia_pedunculata:3.47547)Remijia:3.47547):15.8068,Isertia_rosea:22.7577):8.42972,Exostema_maynense:31.1874):3.69571,(Guettarda_ovo:3.87832,(Chomelia_polyantha:1.93916,Chomelia_tenuiflora:1.93916)Chomelia:1.93916):31.0048):3.41979,Chione_sylvicola:38.3029):17.1354):4.48827,((((Rudgea_bracteata:7.83763,Rudgea_panurensis:7.83763,Rudgea_verticillata:7.83763)Rudgea:16.28023,(Psychotria_crimson:12.0589,Psychotria_mathewsii:12.0589)Psychotria:12.0589):13.5704,(Faramea_anisocalyx:18.8441,Faramea_capillipes:18.8441,Faramea_glandulosa:18.8441,Faramea_multiflora:18.8441,Faramea_parvibractea:18.8441,Faramea_spathacea:18.8441,Faramea_tamberlikiana:18.8441,Faramea_torquata:18.8441)Faramea:18.8441):10.2577,(Coussarea_cephaeloides:19.5015,Coussarea_klugii:19.5015,Coussarea_obliqua:19.5015,Coussarea_paniculata:19.5015,Coussarea_racemosa:19.5015,Coussarea_tenuiflora:19.5015,Coussarea_tigershark:19.5015)Coussarea:28.4444):11.9807):11.6105,((((((Tabernaemontana_sananho:16.529,Macoubea_guianensis:16.529):10.7811,Rauvolfia_polyphylla:27.3102):2.77772,(Parahancornia_peruviana:10.286,((Couma_guianensis:2.48463,Couma_macrocarpa:2.48463)Couma:2.48463,(Lacmellea_lactescens:2.48463,Lacmellea_oblongata:2.48463)Lacmellea:2.48463):5.31678):19.8018):4.28155,(Himatanthus_bracteatus:4.06207,Himatanthus_sucuuba:4.06207,Himatanthus_tarapotensis:4.06207)Himatanthus:30.30727):4.96977,(Aspidosperma_fendleri:8.09132,Aspidosperma_inundatum:8.09132,Aspidosperma_rigidum:8.09132,Aspidosperma_sandwithianum:8.09132,Aspidosperma_schultesii:8.09132,Aspidosperma_spruceanum:8.09132,Aspidosperma_darienense:8.09132,Aspidosperma_desmanthum:8.09132,Aspidosperma_excelsum:8.09132)Aspidosperma:31.2478):5.94869,Strychnos_panurensis:45.2879):26.2492):9.047,((((Tabebuia_moby:9.69934,Tabebuia_serratifolia:9.69934,Tabebuia_vanilla:9.69934)Tabebuia:23.8271,(Jacaranda_copaia:16.7632,Jacaranda_glabra:16.7632)Jacaranda:16.7632):11.9596,((Aegiphila_haughtii:4.68901,Aegiphila_vochy:4.68901)Aegiphila:27.7788,(Vitex_bicolor:14.0586,Vitex_cymosa:14.0586,Vitex_pseudolea:14.0586,Vitex_schunkei:14.0586,Vitex_triflora:14.0586)Vitex:18.4093):13.0182):11.3014,Chionanthus_implicatus:56.7875):23.7966):2.03328,(Cordia_split:16.3066,Cordia_trachyphylla:16.3066,Cordia_ucayaliensis:16.3066,Cordia_alliodora:16.3066,Cordia_collococa:16.3066,Cordia_handsome:16.3066,Cordia_hebeclada:16.3066,Cordia_neat:16.3066,Cordia_nodosa:16.3066)Cordia:66.3107):1.71533,((Solanum_lepidotum:10.2045,Solanum_altissimum:10.2045)Solanum:14.2924,(Cestrum_megalophyllum:12.2485,Cestrum_racemosum:12.2485)Cestrum:12.2485):59.8357):7.67605,Metteniusa_tessmanniana:92.0087):3.27548,(Dendrobangia_boliviana:47.6421,Dendrobangia_multinervia:47.6421)Dendrobangia:47.6421):6.23354,(Schefflera_morototoni:94.0675,((Ilex_inundata:39.4633,Ilex_laureola:39.4633,Ilex_nayana:39.4633)Ilex:39.4633,Discophora_guianensis:78.9265):15.141):7.45023):6.69822,((Symplocos_arechea:65.8009,Styrax_argenteus:65.8009):4.9979,(((((Cariniana_multiflora:3.51855,Couroupita_guianensis:3.51855):6.34326,(((Gustavia_augusta:2.34897,Gustavia_elliptica:2.34897,Gustavia_hexapetala:2.34897,Gustavia_longifolia:2.34897,Gustavia_macarenensis:2.34897)Gustavia:2.34897,(Grias_neuberthii:2.34897,Grias_peruviana:2.34897)Grias:2.34897):3.09105,((Lecythis_oval:2.48578,Lecythis_zabucajo:2.48578)Lecythis:2.48578,(Eschweilera_andina:2.48578,Eschweilera_bracteosa:2.48578,Eschweilera_chartaceifolia:2.48578,Eschweilera_coriacea:2.48578,Eschweilera_decolorans:2.48578,Eschweilera_gigantea:2.48578,Eschweilera_grandiflora:2.48578,Eschweilera_itayensis:2.48578,Eschweilera_juruensis:2.48578,Eschweilera_kaputna:2.48578,Eschweilera_laevicarpa:2.48578,Eschweilera_ovalifolia:2.48578,Eschweilera_parvifolia:2.48578,Eschweilera_rufifolia:2.48578,Eschweilera_tessmannii:2.48578)Eschweilera:2.48578):2.81743):2.07282):1.72195,(Couratari_guianensis:5.79188,Couratari_oligantha:5.79188,Couratari_stellata:5.79188)Couratari:5.79188):51.2687,((Pouteria_pear:4.102,Pouteria_peclargo:4.102,Pouteria_petiolata:4.102,Pouteria_petroleo:4.102,Pouteria_platyphylla:4.102,Pouteria_prominulous:4.102,Pouteria_pubescens:4.102,Pouteria_aubrevillei:4.102,Pouteria_baehniana:4.102,Pouteria_bangii:4.102,Pouteria_reticulata:4.102,Pouteria_rostrata:4.102,Pouteria_bilocularis:4.102,Pouteria_sclerocarpa:4.102,Pouteria_caimito:4.102,Pouteria_simulans:4.102,Pouteria_calistophylla:4.102,Pouteria_suela:4.102,Pouteria_campechiana:4.102,Pouteria_tenuipetiole:4.102,Pouteria_cladantha:4.102,Pouteria_torta:4.102,Pouteria_coriacea:4.102,Pouteria_cuspidata:4.102,Pouteria_trilocularis:4.102,Pouteria_durlandii:4.102,Pouteria_ephedrantha:4.102,Pouteria_filipes:4.102,Pouteria_vernicosa:4.102,Pouteria_final:4.102,Pouteria_glomerata:4.102,Pouteria_gracilis:4.102,Pouteria_guianensis:4.102,Pouteria_hispida:4.102,Pouteria_jariensis:4.102,Pouteria_kaputna:4.102,Pouteria_krukovii:4.102,Pouteria_laevigata:4.102,Pouteria_lobo:4.102,Pouteria_macrophylla:4.102,Pouteria_minga:4.102,Pouteria_multiflora:4.102,Pouteria_nudipetala:4.102,Pouteria_oblanceolata:4.102)Pouteria:4.102,((((Ecclinusa_guianensis:2.25966,Ecclinusa_lanceolata:2.25966,Ecclinusa_ramiflora:2.25966)Ecclinusa:2.25966,Pradosia_atroviolacea:4.51931):1.31931,((Chrysophyllum_amazonicum:2.47845,Chrysophyllum_argenteum:2.47845,Chrysophyllum_bombycinum:2.47845,Chrysophyllum_colombianum:2.47845,Chrysophyllum_cuneifolium:2.47845,Chrysophyllum_lucentifolium:2.47845,Chrysophyllum_manaosense:2.47845,Chrysophyllum_ovale:2.47845,Chrysophyllum_pomiferum:2.47845,Chrysophyllum_prieurii:2.47845,Chrysophyllum_sanguinolentum:2.47845,Chrysophyllum_venezuelanense:2.47845)Chrysophyllum:2.47845,Diploon_cuspidatum:4.95689):0.881727):0.901921,(Elaeoluma_glabrescens:4.42487,(Micropholis_acutangula:2.21244,Micropholis_brochidodroma:2.21244,Micropholis_casiquiarensis:2.21244,Micropholis_egensis:2.21244,Micropholis_guyanensis:2.21244,Micropholis_melinoniana:2.21244,Micropholis_venulosa:2.21244)Micropholis:2.21244):2.31567):1.46346):54.64849):2.10606,((Clavija_procera:56.5962,(Diospyros_artanthifolia:15.6139,Diospyros_capreifolia:15.6139,Diospyros_ekodul:15.6139,Diospyros_nanay:15.6139,Diospyros_pseudoxylopia:15.6139)Diospyros:40.9823):4.9333,Gordonia_fruticosa:61.5295):3.429):5.84025):37.4172):10.3142,((Guapira_relief:3.7277,(Neea_divaricata:1.86385,Neea_fuzzy:1.86385,Neea_garci:1.86385,Neea_green:1.86385,Neea_laxa:1.86385,Neea_macrophylla:1.86385,Neea_psychotrioides:1.86385,Neea_pubiroja:1.86385,Neea_spruceana:1.86385,Neea_stilted:1.86385,Neea_supercrasa:1.86385)Neea:1.86385):79.9405,((Coccoloba_broch:10.438,Coccoloba_coronata:10.438,Coccoloba_densifrons:10.438,Coccoloba_fallax:10.438,Coccoloba_fenestrada:10.438,Coccoloba_immense:10.438,Coccoloba_lehmannii:10.438,Coccoloba_lovely:10.438,Coccoloba_mollis:10.438,Coccoloba_paraensis:10.438,Coccoloba_shoe:10.438)Coccoloba:10.438,(Triplaris_dugandii:5.28731,Triplaris_weigeltiana:5.28731)Triplaris:15.5887):62.7922):34.862):4.42299,(((((Chaunochiton_kappleri:45.5374,Aptandra_tubicina:45.5374):18.2649,Dulacia_candida:63.8024):1.73131,Minquartia_guianensis:65.5337):2.12383,(Schoepfia_lucida:53.7143,(Acanthosyris_annonagustata:46.0818,(Agonandra_peruviana:23.0409,Agonandra_silvatica:23.0409)Agonandra:23.0409):7.63246):13.9432):3.59649,((Heisteria_acuminata:0.819181,Heisteria_nitida:0.819181,Heisteria_spruceana:0.819181)Heisteria:0.819181,Cathedra_acuminata:1.63836):69.6156):51.6992):6.9121):12.0543,(((Panopsis_rubescens:24.5997,(Euplassa_inaequalis:12.2999,Euplassa_occidentalis:12.2999)Euplassa:12.2999):2.91431,Roupala_montana:27.514):106.503,((Meliosma_dudosa:8.6498,Meliosma_herbertii:8.6498,Meliosma_loretoyacuensis:8.6498,Meliosma_palustris:8.6498,Meliosma_polyneura:8.6498,Meliosma_vasquezii:8.6498)Meliosma:8.6498,Ophiocaryon_manausense:17.2996):116.718):7.90218):3.11664,Abuta_grandifolia:145.036):16.0289,(((((((((((((Aniba_megaphylla:0.901336,Aniba_panurensis:0.901336,Aniba_riparia:0.901336,Aniba_rosaeodora:0.901336,Aniba_taubertiana:0.901336,Aniba_williamsii:0.901336,Aniba_guianensis:0.901336,Aniba_hostman:0.901336,Aniba_hostmanniana:0.901336)Aniba:0.901336,(Licaria_aurea:0.901336,Licaria_cannella:0.901336,Licaria_guianensis:0.901336,Licaria_triandra:0.901336)Licaria:0.901336):1.96472,(Ocotea_hirtostyla:1.88369,Ocotea_javitensis:1.88369,Ocotea_laurita:1.88369,Ocotea_lenitae:1.88369,Ocotea_leucoxylon:1.88369,Ocotea_lisagroovy:1.88369,Ocotea_longifolia:1.88369,Ocotea_neblinae:1.88369,Ocotea_negripuberula:1.88369,Ocotea_oblonga:1.88369,Ocotea_obovata:1.88369,Ocotea_olivacea:1.88369,Ocotea_ovalifolia:1.88369,Ocotea_peqsericea:1.88369,Ocotea_quixos:1.88369,Ocotea_scalariformis:1.88369,Ocotea_sp_nov_ceano:1.88369,Ocotea_tessmannii:1.88369,Ocotea_ucayalensis:1.88369,Ocotea_aciphylla:1.88369,Ocotea_amazonica:1.88369,Ocotea_argyrophylla:1.88369,Ocotea_bofo:1.88369,Ocotea_cernua:1.88369,Ocotea_cujumary:1.88369,Ocotea_cuneifolia:1.88369,Ocotea_floribunda:1.88369)Ocotea:1.88369):3.71948,((Endlicheria_sprucei:2.58163,Endlicheria_tschudyana:2.58163,Endlicheria_verticillata:2.58163,Endlicheria_anomala:2.58163,Endlicheria_bracteata:2.58163,Endlicheria_canescens:2.58163,Endlicheria_chalisea:2.58163,Endlicheria_citriodora:2.58163,Endlicheria_directonervia:2.58163,Endlicheria_dori:2.58163,Endlicheria_dysodantha:2.58163,Endlicheria_formosa:2.58163,Endlicheria_gracilis:2.58163,Endlicheria_klugii:2.58163,Endlicheria_krukovii:2.58163,Endlicheria_lorastemon:2.58163,Endlicheria_metallica:2.58163,Endlicheria_miniopacangulo:2.58163,Endlicheria_mishuyacensis:2.58163,Endlicheria_paniculata:2.58163,Endlicheria_ruforamula:2.58163,Endlicheria_sericea:2.58163)Endlicheria:2.58163,((Rhodostemonodaphne_crenaticupula:2.04831,Rhodostemonodaphne_grandis:2.04831,Rhodostemonodaphne_kunthiana:2.04831,Rhodostemonodaphne_napoensis:2.04831,Rhodostemonodaphne_praeclara:2.04831)Rhodostemonodaphne:2.04831,(Nectandra_matthewsii:2.04831,Nectandra_maynensis:2.04831,Nectandra_membranacea:2.04831,Nectandra_oppositifolia:2.04831,Nectandra_parviflora:2.04831,Nectandra_paucinervia:2.04831,Nectandra_pearcei:2.04831,Nectandra_purpurea:2.04831,Nectandra_reticulata:2.04831,Nectandra_rojaretic:2.04831,Nectandra_viburnoides:2.04831,Nectandra_anib:2.04831,Nectandra_canescens:2.04831,Nectandra_cissiflora:2.04831,Nectandra_coeloclada:2.04831,Nectandra_crasa:2.04831,Nectandra_crassiloba:2.04831,Nectandra_cuneatocordata:2.04831,Nectandra_fragrans:2.04831,Nectandra_gracilis:2.04831,Nectandra_lineata:2.04831)Nectandra:2.04831):1.06665):2.32361):1.58092,Persea_pseudofasciculata:9.0678):1.40167,(Aiouea_brasiliensis:5.23473,Aiouea_impressa:5.23473)Aiouea:5.23473):2.46786,(Cinnamomum_napoense:6.46866,Cinnamomum_triplinerve:6.46866)Cinnamomum:6.46866):21.3413,((Chlorocardium_subopo:8.64814,Chlorocardium_venenosum:8.64814)Chlorocardium:8.64814,(Beilschmiedia_costaricensis:8.64814,Beilschmiedia_pendula:8.64814)Beilschmiedia:8.64814):16.9823):38.8557,(Mollinedia_ovata:36.5672,Mollinedia_panther:36.5672)Mollinedia:36.5672):19.3953,(Siparuna_cervicornis:41.7562,Siparuna_cuspidata:41.7562,Siparuna_decipiens:41.7562,Siparuna_macrotepala:41.7562,Siparuna_pube:41.7562)Siparuna:50.7734):21.9075,((((Osteophloeum_platyspermum:6.32591,(Iryanthera_crassifolia:3.16295,Iryanthera_grandis:3.16295,Iryanthera_hostmannii:3.16295,Iryanthera_juruensis:3.16295,Iryanthera_laevis:3.16295,Iryanthera_lancifolia:3.16295,Iryanthera_macrophylla:3.16295,Iryanthera_tessmannii:3.16295,Iryanthera_ulei:3.16295)Iryanthera:3.16295):2.91038,(Compsoneura_capitellata:4.61815,Compsoneura_sprucei:4.61815,Compsoneura_ulei:4.61815)Compsoneura:4.61815):4.28565,(Virola_mollissima:6.76097,Virola_multinervia:6.76097,Virola_obovata:6.76097,Virola_parvifolia:6.76097,Virola_pavonis:6.76097,Virola_peruviana:6.76097,Virola_sebifera:6.76097,Virola_surinamensis:6.76097,Virola_theiodora:6.76097,Virola_decorticans:6.76097,Virola_divergens:6.76097,Virola_duckei:6.76097,Virola_elongata:6.76097,Virola_flexuosa:6.76097,Virola_marlenei:6.76097)Virola:6.76097):76.0803,(((((((((Annona_ambotay:4.59189,Annona_corky:4.59189,Annona_dolichopetala:4.59189,Annona_duckei:4.59189,Annona_edulis:4.59189,Annona_glomerulifera:4.59189,Annona_helosioides:4.59189,Annona_papilionella:4.59189)Annona:14.1725,(Diclinanona_calycina:9.38217,Diclinanona_tessmannii:9.38217)Diclinanona:9.38217):12.1668,((Xylopia_aromatica:14.3484,Xylopia_calophylla:14.3484,Xylopia_excellens:14.3484,Xylopia_ligustrifolia:14.3484,Xylopia_multiflora:14.3484,Xylopia_parviflora:14.3484,Xylopia_pubescent:14.3484,Xylopia_sericea:14.3484,Xylopia_surinamensis:14.3484)Xylopia:14.3484,(Fusaea_longifolia:15.563,(Duguetia_flagellaris:7.78151,Duguetia_hadrantha:7.78151,Duguetia_odorata:7.78151,Duguetia_quitarensis:7.78151,Duguetia_spixiana:7.78151,Duguetia_surinamensis:7.78151)Duguetia:7.78151):13.1339):2.23426):1.5832,((Guatteria_recurvisepala:1.1707,Guatteria_schomburgkiana:1.1707,Guatteria_streambed:1.1707,Guatteria_asplundiana:1.1707,Guatteria_brevicuspis:1.1707,Guatteria_citriodora:1.1707,Guatteria_diana:1.1707,Guatteria_elata:1.1707,Guatteria_gentryi:1.1707,Guatteria_glaberrima:1.1707,Guatteria_guianensis:1.1707,Guatteria_hispida:1.1707,Guatteria_impreso:1.1707,Guatteria_megalophylla:1.1707,Guatteria_multivenia:1.1707,Guatteria_puncticulata:1.1707)Guatteria:1.1707,Guatteriopsis_ramiflora:2.3414):30.173):4.03176,((Trigynaea_duckei:8.83746,Trigynaea_triplinervis:8.83746)Trigynaea:8.83746,Porcelia_mediocris:17.6749):18.8712):2.77877,Tetrameranthus_globuliferum:39.3249):3.37008,(((((Oxandra_euneura:3.3173,Oxandra_mediocris:3.3173,Oxandra_riedeliana:3.3173,Oxandra_xylopioides:3.3173)Oxandra:3.3173,(Pseudomalmea_diclina:2.13029,Klarobelia_napoensis:2.13029):4.50431):2.43498,Ruizodendron_ovale:9.06958):7.14733,Pseudoxandra_polyphleba:16.2169):2.151,(Unonopsis_elegantissima:1.83423,Unonopsis_floribunda:1.83423,Unonopsis_qca:1.83423,Unonopsis_spectabilis:1.83423,Unonopsis_stipitata:1.83423)Unonopsis:16.5337):24.3271):8.30332,(Anaxagorea_brevipes:25.4991,Anaxagorea_dolichocarpa:25.4991,Anaxagorea_phaeocarpa:25.4991)Anaxagorea:25.4991):21.8857,Talauma_fine:72.88395):16.7183):24.8349):7.45859,(Piper_bellidifolium:49.2426,Piper_reticulatum:49.2426)Piper:72.6532):19.7439,(((Phytelephas_tenuicaulis:13.31054,((((Astrocaryum_chambira:1.64629,Astrocaryum_murumuru:1.64629)Astrocaryum:7.66987,(Attalea_butyracea:1.37062,Attalea_maripa:1.37062)Attalea:7.94553):1.13237,(Euterpe_precatoria:5.5405,(Oenocarpus_bataua:2.77025,Oenocarpus_kampa:2.77025,Oenocarpus_mapora:2.77025)Oenocarpus:2.77025):4.90801):1.65942,(((Wettinia_drudei:1.14755,Wettinia_longipetala:1.14755,Wettinia_maynensis:1.14755)Wettinia:2.03222,Iriartea_deltoidea:3.17977):1.50335,(Socratea_exorrhiza:2.34156,Socratea_rostrata:2.34156)Socratea:2.34156):7.42483):1.2026):4.64011,Chelyocarpus_ulei:17.9507):40.8719,(Mauritia_flexuosa:2.63711,Mauritiella_armata:2.63711):56.1855):82.817):19.4255);
